# Limitations of the p16-3MR mouse model for detecting and eliminating senescent cells

**DOI:** 10.1038/s44319-026-00802-8

**Published:** 2026-05-28

**Authors:** Nozomi Hori, Shimpei Kawamoto, Ken Uemura, Yumi Kinugasa-Katayama, Yumiko Okumura, Kentaro Tanaka, Jeong Hoon Park, Masahiro Wakita, Daisuke Motooka, Naoko Ohtani, Eiji Hara

**Affiliations:** 1 Department of Molecular Biology, Research Institute for Microbial Diseases, The University of Osaka, Suita, 565-0871 Japan; 2 Department of Aging Biology, Institute of Development, Aging and Cancer, Tohoku University, Sendai, 980-8575 Japan; 3 Organization for Advanced Studies, Tohoku University, Sendai, 980-8575 Japan; 4 NGS Core Facility, Research Institute for Microbial Diseases, The University of Osaka, Suita, 565-0871 Japan; 5 Department of Pathophysiology, Graduate School of Medicine, Osaka Metropolitan University, Osaka, 545-8585 Japan; 6 Center for Infectious Diseases Education and Research, The University of Osaka, Suita, 565-0871 Japan; 7 Immunology Frontier Research Center, The University of Osaka, Suita, 565-0871 Japan

**Keywords:** Methods & Resources, Molecular Biology of Disease

## Abstract

The *p16-3MR* mouse model, designed to express Renilla luciferase, mRFP, and herpes simplex virus 1 thymidine kinase (HSV-TK) under the *p16*^*INK4a*^ promoter, has been widely used to visualize and ablate senescent cells in vivo, but our analyses revealed critical limitations. Bioluminescence signals in *p16-3MR* mice were extremely weak and virtually indistinguishable from those of wild-type mice injected with coelenterazine-h, indicating that previously reported signals largely reflected substrate background rather than authentic reporter expression. Signal intensity remained unchanged with aging, doxorubicin treatment, or cutaneous wound healing, failing to replicate earlier observations. Furthermore, RFP signals were undetectable in senescent fibroblasts from *p16-3MR* mice, and senescent cells were not eliminated by ganciclovir treatment, suggesting poor expression and lack of functional activity of the mRFP and HSV-TK transgenes. These results demonstrate functional deficiencies in all three transgenes, highlighting the importance of using wild-type controls and calling for careful reevaluation of studies employing this system.

## Synopsis

The p16-3MR mouse model is widely used to detect and eliminate senescent cells in vivo. We here report that its bioluminescence signals primarily reflect substrate-dependent background and do not definitively report endogenous p16^INK4a^ expression.

- Bioluminescence signals in p16-3MR mice were comparable to wild-type mice, indicating that reported signals largely reflected coelenterazine-h substrate background.
- No age-associated increase in bioluminescence and no transient signal induction during cutaneous wound healing were observed, failing to replicate earlier findings.
- RFP was undetectable and ganciclovir did not eliminate senescent cells in vitro, demonstrating lack of functional mRFP and HSV-TK transgene activity.
- Ganciclovir suppressed macrophage numbers independently of HSV-TK, raising the possibility that reported phenotypic changes may reflect off-target effects.

## Abstract (web summary)

The p16-3MR mouse model is widely used to detect and eliminate senescent cells in vivo. We here report that its bioluminescence signals primarily reflect substrate-dependent background and do not definitively report endogenous p16^INK4a^ expression.

## Graphical abstract

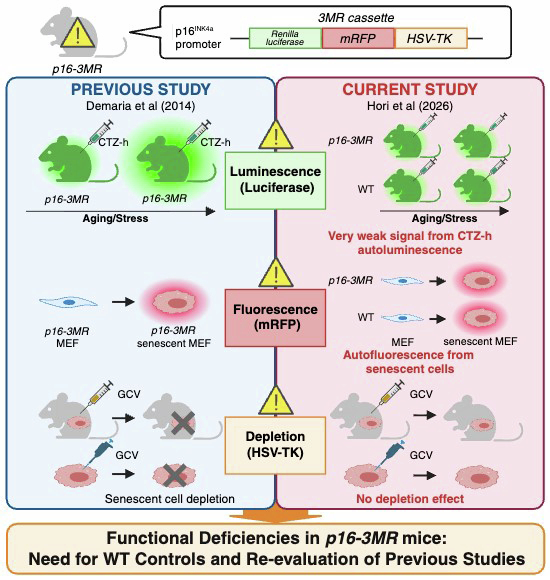

## Introduction

Cellular senescence is a state of irreversible cell cycle arrest induced by various stresses, including telomere shortening, oncogene activation, radiation, ultraviolet light, DNA-damaging agents, and oxidative stress, all of which may pose a risk of tumorigenesis. Consequently, cellular senescence has long been regarded as an important tumor suppression mechanism (Campisi and d’Adda di Fagagna, 2007; Collado and Serrano, 2010; He and Sharpless, 2017). However, senescent cells do not undergo immediate cell death and therefore accumulate in various tissues with advancing age. Importantly, senescent cells acquire a characteristic known as the senescence-associated secretory phenotype (SASP), which involves the secretion of numerous pro-inflammatory factors (Acosta et al, 2008; Coppe et al, 2008; Kuilman et al, 2008). As a result, it has become increasingly evident that the accumulation of senescent cells in tissues contributes to chronic inflammation and promotes the development of various aging-related diseases, including cancer (Chan and Narita, 2019; Wang et al, 2022). Accordingly, reducing the burden of senescent cells in vivo has been proposed as a potential strategy to delay the onset of age-associated diseases (Baker et al, 2016; Baker et al, 2011). However, given the multifaceted roles of cellular senescence (Grosse et al, 2020; Rodier and Campisi, 2011), a precise understanding of their in vivo functions is essential for the rational and safe targeting of senescent cells. To achieve this goal, a reliable mouse model that allows for both the in vivo detection and elimination of senescent cells is required. Several such mouse models rely on the *p16*^*INK4a*^ or *p21*^*Waf1/Cip1/Sdi1*^ gene promoters, which are active in senescent cells (Baker et al, 2011; Burd et al, 2013; Demaria et al, 2014; Grosse et al, 2020; Hashimoto et al, 2016; Haston et al, 2023; Ohtani et al, 2007; Wang et al, 2021; Yamakoshi et al, 2009; Zhao et al, 2024). Among these models, the *p16-3MR* mouse model is one of the most widely used (Demaria et al, 2014).

The *p16-3MR* transgenic mouse (Demaria et al, 2014) harbors a bacterial artificial chromosome (BAC) containing approximately 50 kb of the mouse *p16*^*INK4a*^ gene locus, and the *p16*^*INK4a*^ gene promoter drives the expression of the 3MR (trimodality reporter) fusion protein, which contains the functional domains of a synthetic Renilla luciferase (Rluc), monomeric red fluorescent protein (mRFP), and truncated herpes simplex virus 1 thymidine kinase (HSV-TK) (Ray et al, 2004). This design enables both the visualization and conditional ablation of senescent cells in vivo (Demaria et al, 2014). Using this mouse model, Campisi’s group, in collaboration with ours, previously reported that senescent fibroblasts and endothelial cells transiently appear at early stages of cutaneous wound healing, where they promote tissue repair by inducing myofibroblast differentiation through the secretion of platelet-derived growth factor AA (PDGF-AA), a SASP component (Demaria et al, 2014). To our knowledge, this was the first study to demonstrate the beneficial effects of SASP-mediated senescence on tissue homeostasis. In that study, our group contributed data obtained using double-knockout (DKO) mice lacking both the *p16*^*INK4a*^ and *p21*^*Waf1/Cip1/Sdi1*^ genes (Takeuchi et al, 2010), and Campisi’s group employed the *p16-3MR* mouse model. The combined results were jointly reported (Demaria et al, 2014). However, after obtaining *p16-3MR* mice from Campisi’s group and examining them in our own laboratory, we identified several critical problems with this mouse model. Given its widespread use in the field of senescence research, we consider it essential to disclose these issues and raise awareness of its potential limitations. We therefore report here the principal limitations of the *p16-3MR* mouse model.

## Results and discussion

**Figure 1:**
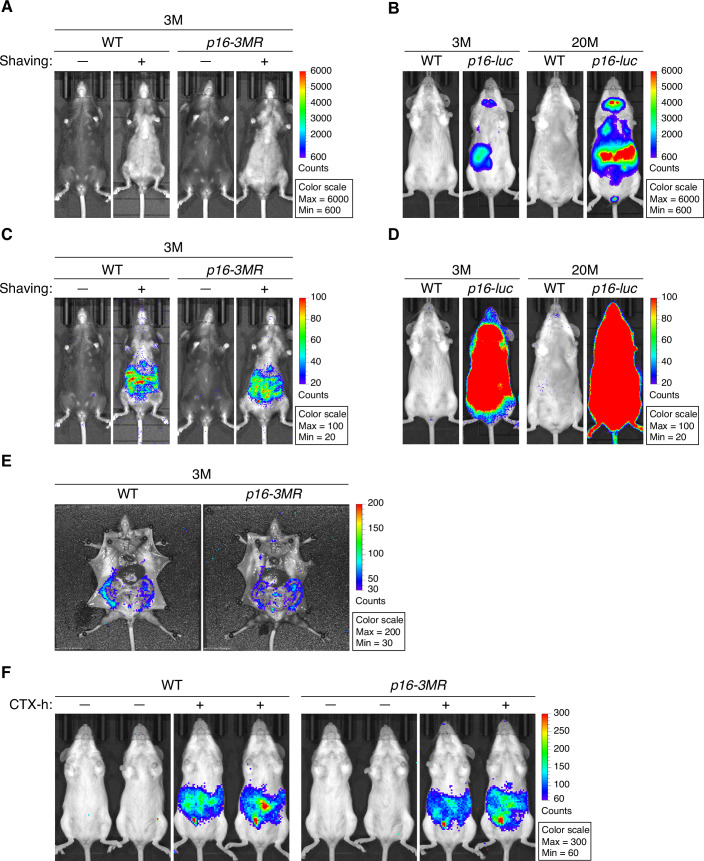
Background luminescence of coelenterazine-h in *p16-3MR* mice. (**A**–**E**) Three-month-old female WT and *p16-3MR* mice (**A**,** C**), and 3- and 20-month-old female albino WT and *p16-luc* mice (**B**,** D**) were injected with coelenterazine-h (CTZ-h; 15 µg) or D-luciferin (75 mg/kg), respectively, and subjected to in vivo bioluminescence imaging (BLI) using the IVIS imaging system. For *p16-3MR* mice (**A**,** C**), BLI was performed before and after shaving the black fur (left and right panels, respectively). Panel (**E**) shows imaging performed following laparotomy. The same imaging data are displayed using different color scale intensities: minimum and maximum thresholds were set at 600 and 6,000 counts for (**A**,** B**), and 20 and 100 counts for (**C**,** D**). (**F**) Three-month-old albino female WT and *p16-3MR* mice underwent BLI before and after CTZ-h administration. Genotyping and treatment status for each BLI image are indicated in the top panel. For BLI acquisition, the binning setting was set to “medium” for (**A**–**D**) and “large” for (**E**,** F**). Color bars represent signal intensity (counts) corresponding to the indicated minimum and maximum thresholds. Experiments were independently repeated at least three times with similar results.

The use of albino mice with white fur is generally recommended for in vivo bioluminescence imaging (BLI) to minimize the attenuation of luminescent signals by dark fur (Ji et al, 2020). Additionally, in dark-furred mice, the skin darkens when the hair cycle enters the anagen phase, further obstructing luminescent signals (Curtis et al, 2011). Therefore, if dark-furred mice must be used, shaving the fur and verifying the absence of skin darkening are strongly recommended for in vivo BLI, particularly when detecting low-intensity luminescent signals. However, we noted that Demaria et al, (2014) performed in vivo BLI without shaving the black fur. To replicate their experimental conditions, we conducted non-invasive in vivo BLI in 3-month-old *p16-3MR* mice following the same protocol, in which imaging was performed without fur removal after intraperitoneal injection of coelenterazine-h (CTZ-h), the substrate for Renilla luciferase (Rluc). Because the user manual for the IVIS imaging system (Revvity Inc.) indicates that luminescence signals below 600 counts in mice are considered background noise, we performed in vivo BLI using a detection threshold of ≥600 counts. Under these conditions, no bioluminescence signal was detectable in *p16-3MR* mice, even after shaving the black fur (Fig. 1A), whereas robust signals were readily detected in *p16-luc* mice expressing firefly luciferase under the human *p16*^*INK4a*^ promoter (Kawamoto et al, 2023) (Fig. 1B). Notably, in Demaria et al, (2014), the minimum value of the color scale (i.e., the detection threshold) was set to approximately 10–30 counts, a range sufficiently low to capture background noise as apparent signals, as indicated by the color bars in their figures. Consistent with this observation, lowering the detection threshold to 20 counts enabled detection of luminescence in shaved black-furred *p16-3MR* mice (Fig. 1C), whereas signals in *p16-luc* mice were saturated and exceeded the dynamic range of detection (Fig. 1D).

Given that the luminescence intensity observed, even after shaving the fur, remained extremely low (below 100 counts), and that CTZ-h is known to generate weak luminescent signals independently of Rluc through reactions with albumin (Zhao et al, 2004) or superoxide (Bronsart et al, 2016), we reasoned that the luminescence observed in *p16-3MR* mice likely reflected CTZ-h–derived background luminescence rather than genuine Rluc-dependent luminescence. Because coelenterazine auto-oxidation produces the same luminescent emitter (coelenteramide) as the Rluc reaction (Zhao et al, 2004), luciferase-dependent and independent signals cannot be reliably distinguished by spectral filtering. We therefore compared luminescence signals side-by-side in shaved *p16-3MR* mice and non-transgenic wild-type (WT) C57BL/6 mice. Weak luminescent signals were detected in WT mice at levels comparable to those observed in shaved *p16-3MR* mice (Fig. 1C). Furthermore, following the injection of CTZ-h, we performed in vivo BLI by laparotomy on both *p16-3MR* mice and wild-type (WT) mice. The luminescent signals detected in the two strains were comparable (Fig. 1E), suggesting that the luminescence observed in *p16-3MR* mice is likely attributable to background luminescence from CTZ-h, generated by serum albumin and/or superoxide, rather than to Rluc expression, at least in this experimental setting. To exclude the possibility that shaving induced unexpected stress leading to luminescent signals (Wright et al, 2017), we conducted in vivo BLI using albino C57BL/6 mice crossed with *p16-3MR* mice to produce white-furred *p16-3MR* mice. Using these mice, we confirmed that both WT and *p16-3MR* mice emitted similarly weak luminescence signals upon CTZ-h administration, even without shaving their fur (Fig. 1F), indicating that the luminescence observed in *p16-3MR* mice most likely reflects CTZ-h–derived background luminescence. These results reaffirm that in vivo BLI is best performed using white-furred mice. If black-furred mice are used, the fur should be shaved to enable accurate detection of luminescent signals, particularly when detecting weak signals (Ji et al, 2020).

**Figure 2:**
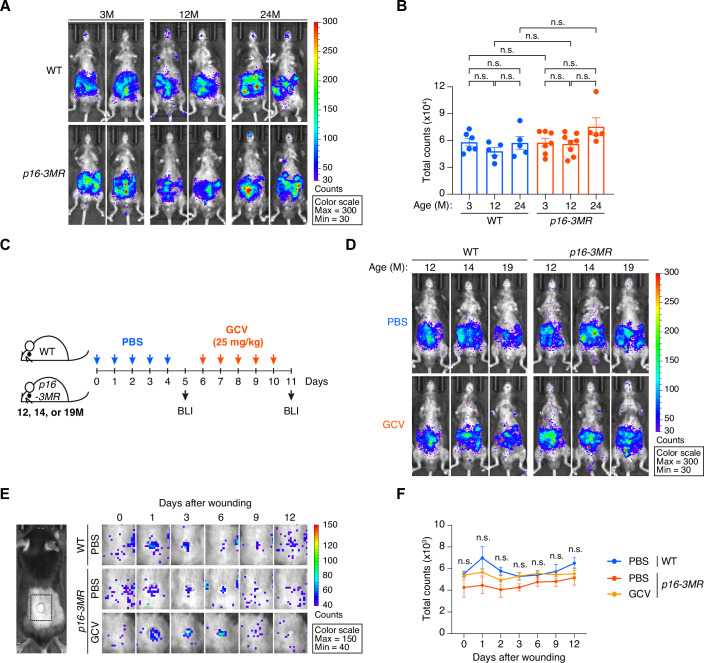
Lack of increased luminescence with aging or wound healing in *p16-3MR* mice. (**A**,** B**) Male WT and *p16-3MR* mice were shaved and subjected to BLI at 3, 12, and 24 months of age. Representative BLI images are shown in (**A**), and quantification of bioluminescence intensity is shown in (**B**). (**C**,** D**) Male WT and *p16-3MR* mice at 12, 14, or 19 months of age were intraperitoneally injected with PBS (vehicle control) or ganciclovir (GCV; 25 mg/kg). (**C**) shows the experimental timeline corresponding to the data in (**D**). In (**D**), representative BLI images from the same mouse are shown one day after the final injection, with images obtained following PBS and GCV administration displayed in the upper and lower panels, respectively. (**E**,** F**) Two-month-old male WT and *p16-3MR* mice were wounded on the dorsal skin using a 6-mm biopsy punch and treated with PBS or GCV by intraperitoneal injection from day 1 to day 6 post-injury. BLI was performed on the indicated days. Representative BLI images of the wound site are shown in (**E**), and quantification of bioluminescence intensity in the wound area is shown in (**F**). All imaging procedures were performed with binning set to “large”. Color bars represent signal intensity (counts) corresponding to the indicated minimum and maximum thresholds. The sample size (n) represents the number of biologically independent animals (**B**, *n* = 5–8; **F**, *n* = 4–5). Data are presented as mean ± s.e.m. Statistical significance was determined using two-way ANOVA followed by Tukey’s multiple-comparison test (**B**,** F**). No statistically significant differences were observed among the groups in (**B**,** F**); n.s. not significant. Experiments were independently repeated at least twice with similar results. Source data are available online for this figure.

An important question is whether the reported age-associated increase in bioluminescence signals in *p16-3MR* mice (Figure S2 in Demaria et al, 2014) is reproducible. To address this question, we performed in vivo BLI on *p16-3MR* and WT mice after shaving their fur. However, in contrast to *p16-luc* mice (Fig. 1B), we detected only background CTZ-h–derived signals in aged *p16-3MR* mice, with no age-associated increase and at levels comparable to those observed in WT mice (Fig. 2A,B). Furthermore, Demaria et al, (2014) reported that ganciclovir (GCV) administration to 24-month-old *p16-3MR* mice led to a marked reduction in bioluminescent signals (Fig. 2C in Demaria et al, 2014). However, we were unable to reproduce this result (Fig. 2C,D). One possible explanation for this discrepancy may lie in differences in experimental design. Specifically, comparisons based on separate PBS- and GCV-treated groups (Demaria et al, 2014) are susceptible to inter-individual variability, particularly when bioluminescence signals are extremely weak and near the detection limit. By directly comparing signals in the same animals before and after GCV treatment, our within-subject design minimizes such variability and did not reveal a consistent GCV-dependent signal reduction (Fig. 2C,D). Moreover, because black fur was not shaved in the study by Demaria et al, (2014), we suspect that the apparent age-associated increase in bioluminescent signals observed in some *p16-3MR* mice (Figure S2 in Demaria et al, 2014) may largely reflect CTZ-h–derived background luminescence, which could become more readily detectable as body hair grays, thins, or is lost during aging (Matsumura et al, 2021). We also tested whether the bioluminescent signal indicative of *p16*^*INK4a*^ expression is transiently induced during skin wound healing at day 6 post-wounding in *p16-3MR* mice, as reported in Demaria et al, (2014) (their Fig. 3A,B), and whether it is suppressed by GCV administration. However, unlike the data reported by Demaria et al, (2014), the bioluminescent signal in our *p16-3MR* skin wound-healing model remained extremely low. We observed neither a transient increase at day 6 post-wounding nor any significant change following GCV treatment, and signal levels were comparable between *p16-3MR* and WT mice (Fig. 2E,F). These observations suggest that the detected signals are most consistent with background noise.

**Figure 3:**
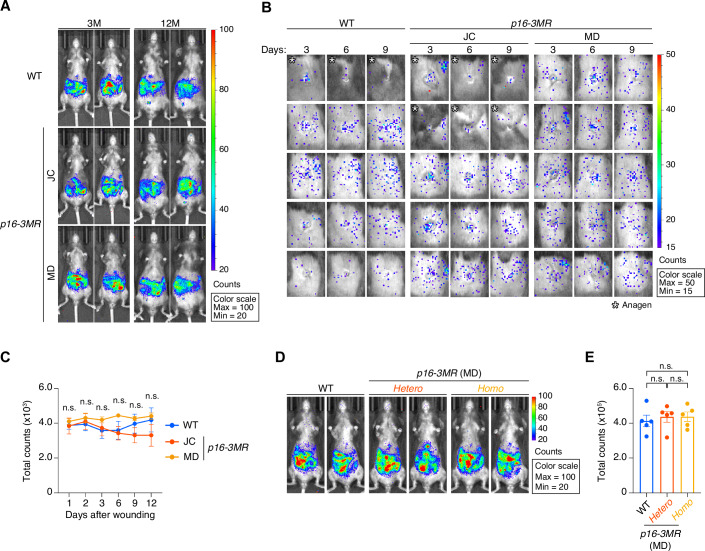
No differences between Campisi- and Demaria-derived *p16-3MR* mice. (**A**–**C**) BLI was performed on WT mice and *p16-3MR* mice obtained from the Campisi and Demaria laboratories. The same female WT and *p16-3MR* mice were subjected to BLI at 3 and 12 months of age (**A**). Three-month-old female WT and *p16-3MR* mice were wounded on the dorsal skin using a 6-mm biopsy punch and subjected to BLI at days 3, 6, and 9 post-injury (**B**,** C**). Representative BLI images of the wound site are shown in (**B**), and quantification of bioluminescence intensity in the wound area is shown in (**C**). Asterisks in (**B**) indicate mice in the anagen phase of the hair cycle. (**D**,** E**) Female WT, *p16-3MR* (MD) heterozygous, and *p16-3MR* (MD) homozygous mice derived from the same litter were shaved and subjected to BLI at 2 months of age. Representative BLI images are shown in (**D**), and quantification of bioluminescence intensity is shown in (**E**). Imaging was performed with binning set to “medium”. Color bars represent signal intensity (counts) corresponding to the indicated minimum and maximum thresholds. The sample size (*n*) represents the number of biologically independent animals (**C**,** E**; *n* = 5). Data were presented as mean ± s.e.m. Statistical significance was determined by one-way ANOVA followed by Tukey’s multiple-comparison test (**C**,** E**). No statistically significant differences were detected (**C**,** E**); n.s. not significant. Experiments were independently repeated at least three times with similar results. Source data are available online for this figure.

In light of our inability to reproduce the findings reported for *p16-3MR* mice in Demaria et al, (2014), we hypothesized that the strain maintained in our laboratory might differ either genetically or functionally from that used by Demaria’s group. Therefore, we obtained *p16-3MR* mice directly from Demaria’s laboratory and conducted side-by-side comparative experiments with the *p16-3MR* mice obtained from Campisi’s group. First, whole-genome sequencing of the *p16-3MR* mice obtained from the Campisi and Demaria laboratories, hereafter referred to as *p16-3MR* (JC) and *p16-3MR* (MD), respectively, revealed no differences in the *3MR* transgene sequences. Next, we performed in vivo BLI on shaved 3-month-old and 12-month-old mice, but detected no significant differences in signal intensities among the *p16-3MR* (JC), *p16-3MR* (MD), and WT mice (Fig. 3A). Furthermore, we observed no transient increase in bioluminescence during skin wound healing at day 6 post-wounding in 3-month-old *p16-3MR* (JC), *p16-3MR* (MD), or WT mice (Fig. 3B,C). We also tested whether the homozygosity of the reporter transgene would enhance the bioluminescent signal. However, 2-month-old *p16-3MR* (MD) mice showed no significant differences compared with WT littermates, irrespective of whether the transgene was present in the heterozygous or homozygous state (Fig. 3D,E). Although no differences were identified in the *3MR* transgene sequence between the two *p16-3MR* strains, functional alterations unrelated to primary transgene sequence could still be considered. One such possibility is epigenetic silencing of the transgene over successive generations, a phenomenon known to occur in certain transgenic mouse lines. However, it is noteworthy that the bioluminescence signal intensities of *p16-3MR* mice in the original study were already near the detection limit when first reported (Demaria et al, 2014). Specifically, the reported signal intensities ranged from approximately 10–50 counts (Fig. 2A in Demaria et al, 2014), 20–150 counts (Fig. 2C in Demaria et al, 2014), 20–80 counts (Fig. 3B in Demaria et al, 2014), and 10–50 counts (Fig. S2D in Demaria et al, 2014). These values are highly comparable to the signal intensities observed in our current study, as indicated by the color scales shown in both our figures and those of Demaria et al, (2014), and fall within the noise range according to the instruction manual of the IVIS imaging system (Revvity Inc.). Thus, even in the original study (Demaria et al, 2014), the reported bioluminescence signals were well below the recommended threshold for reliable detection. Collectively, these observations indicate that the *3MR* transgene was expressed at levels near the detection limit of the IVIS system at the time of the original publication and has not changed substantially over subsequent generations.

**Figure 4:**
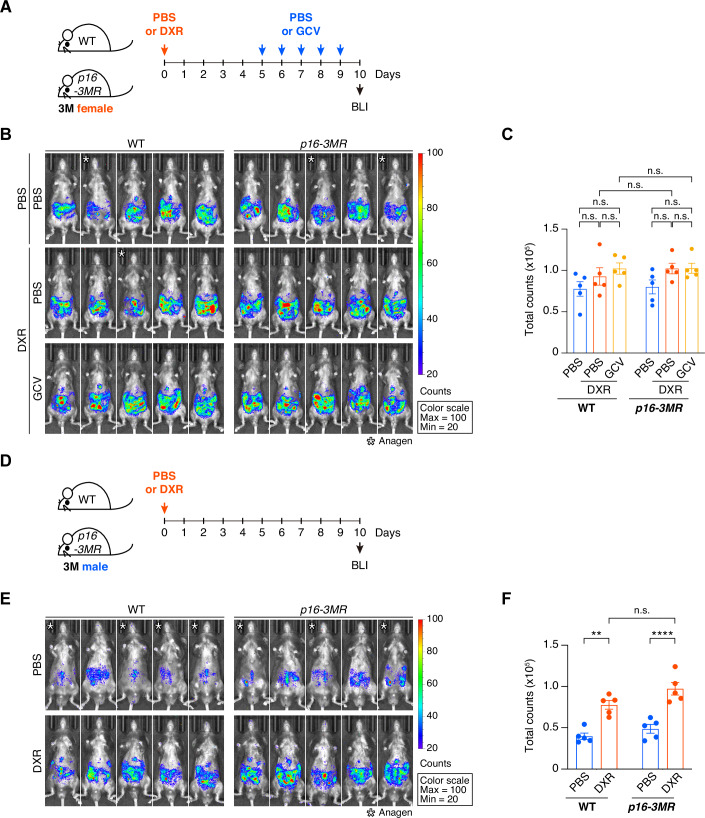
No increase in luminescence signals after DXR administration in *p16-3MR* mice. (**A**–**C**) Three-month-old female WT and *p16-3MR* (MD) mice were intraperitoneally injected with PBS (vehicle control) or doxorubicin (DXR; 10 mg/kg) on day 0. From days 5 to 9, mice received daily intraperitoneal injections of PBS (vehicle control) or ganciclovir (GCV; 25 mg/kg), and bioluminescence imaging (BLI) was performed on day 10 (**A**). Representative BLI images of WT and *p16-3MR* mice on day 10 are shown in the left and right panels, respectively (**B**). Treatment conditions (PBS, DXR, or GCV) are indicated in the labels on the far left; asterisks mark mice in the anagen phase of the hair cycle. Quantification of abdominal bioluminescence intensity is shown in (**C**). (**D**–F) Three-month-old male WT and *p16-3MR* mice were intraperitoneally injected with PBS (vehicle control) or DXR (10 mg/kg) on day 0 and subjected to BLI on day 10 (**D**). Representative BLI images of WT (left) and *p16-3MR* mice (right) are shown in (**E**). Treatment conditions are indicated in the labels on the far left; asterisks mark mice in the anagen phase of the hair cycle. Quantification of abdominal bioluminescence intensity is shown in (**F**). All imaging was performed with binning set to “medium”. Color bars represent signal intensity (counts) corresponding to the indicated minimum and maximum thresholds. The sample size (n) represents the number of biologically independent animals (**C**,** F**; *n* = 5 per group). Data were presented as mean ± s.e.m. Statistical significance was determined by two-way ANOVA followed by Šídák’s multiple-comparison test (**C**,** F**). No statistically significant differences were observed in (**C**). (**F**) **P = 0.0013 (WT; PBS vs. DXR), ****P < 0.0001 (*p16-3MR*; PBS vs. DXR), and *P* = 0.1460 (DXR; WT vs. *p16-3MR*). **P < 0.01, ****P < 0.0001; n.s., not significant. Experiments were independently repeated at least twice with similar results. Source data are available online for this figure.

**Figure EV1:**
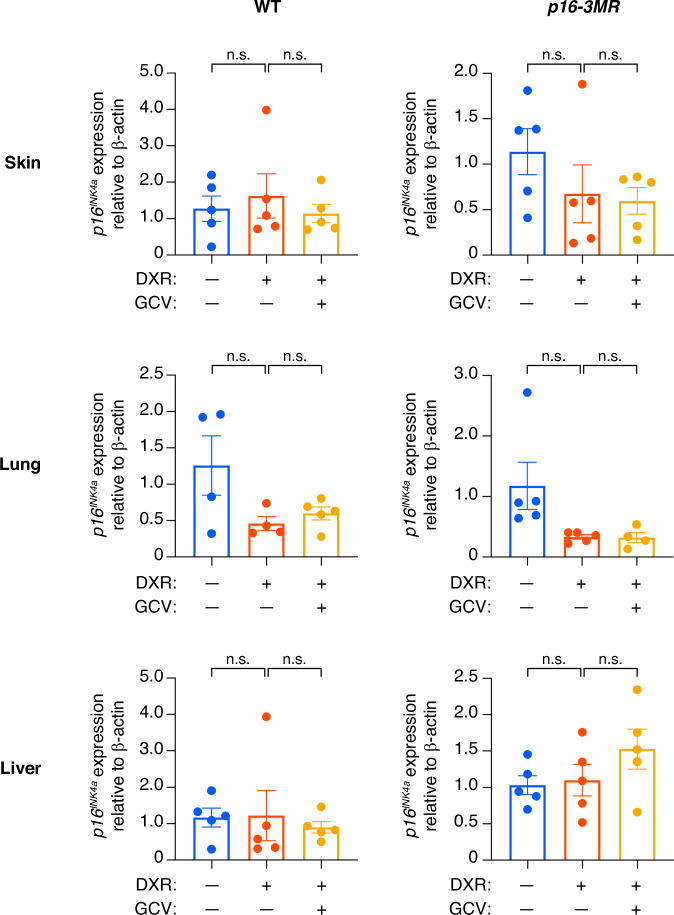
No change in *p16*^*INK4a*^ expression in *p16-3MR* tissues after DXR or GCV treatment. Three-month-old female WT and *p16-3MR* (MD) mice (the same cohort used in Fig. 4A–C) were intraperitoneally injected with PBS (vehicle control) or doxorubicin (DXR; 10 mg/kg) on day 0. From days 5 to 9, mice received daily intraperitoneal injections of PBS (vehicle control) or ganciclovir (GCV; 25 mg/kg). On day 10, *p16*^*INK4a*^ expression levels in the skin, lung, and liver were analyzed by RT-qPCR. Data were presented as relative *p16*^*INK4a*^ expression normalized to the untreated control group (DXR−/GCV−). The sample size (*n*) represents the number of biological replicates (*n* = 4–5). Data were presented as mean ± s.e.m. Statistical significance was determined by one-way ANOVA followed by Šídák’s multiple-comparison test. n.s. not significant. Experiments were independently repeated at least twice with similar results.

Demaria et al, (2017) reported that administration of the DNA-damaging agent doxorubicin (DXR) to *p16-3MR* mice increased bioluminescent signals, which were subsequently reduced by GCV treatment. To assess the reproducibility of these findings, we examined *p16-3MR* (MD) mice under the same experimental conditions, using WT littermates as negative controls. However, as shown in Fig. 4A–C, no statistically significant changes in bioluminescent signal intensity were detected in *p16-3MR* (MD) mice following DXR or GCV treatment, and the overall signal levels were comparable to those observed in WT mice. Consistently, RT-qPCR analysis revealed no significant changes in endogenous *p16*^*INK4a*^ expression in skin, lung, or liver tissues (Fig. EV1). Although a slight increase in bioluminescence was observed following DXR treatment in *p16-3MR* (MD) mice, this trend was not statistically significant and, importantly, a similar increase was also detected in WT mice (Fig. 4B,C), arguing against a specific induction of Rluc expression and instead suggesting substrate-dependent background signals. During these experiments, we noted a reduced frequency of mice in the anagen phase of the hair cycle among DXR-treated animals (Fig. 4B), consistent with previous reports that DXR disrupts hair cycling and induces alopecia (Amoh et al, 2007). As described above, even when black fur is shaved, the skin darkens during anagen, thereby attenuating luminescence transmission and potentially affecting BLI measurements (Curtis et al, 2011). Because male mouse skin is approximately 40% thicker than that of females (Azzi et al, 2005), we considered whether this effect might be more pronounced in males. Indeed, bioluminescent signals were modestly lower in males than in females, and a slight but statistically significant increase was detected in shaved male *p16-3MR* (MD) mice 10 days after DXR treatment (Fig. 4D–F). However, an equivalent increase was again observed in WT mice, indicating that these changes were unlikely to reflect upregulated Rluc activity and were instead most consistent with background noise.

**Figure 5:**
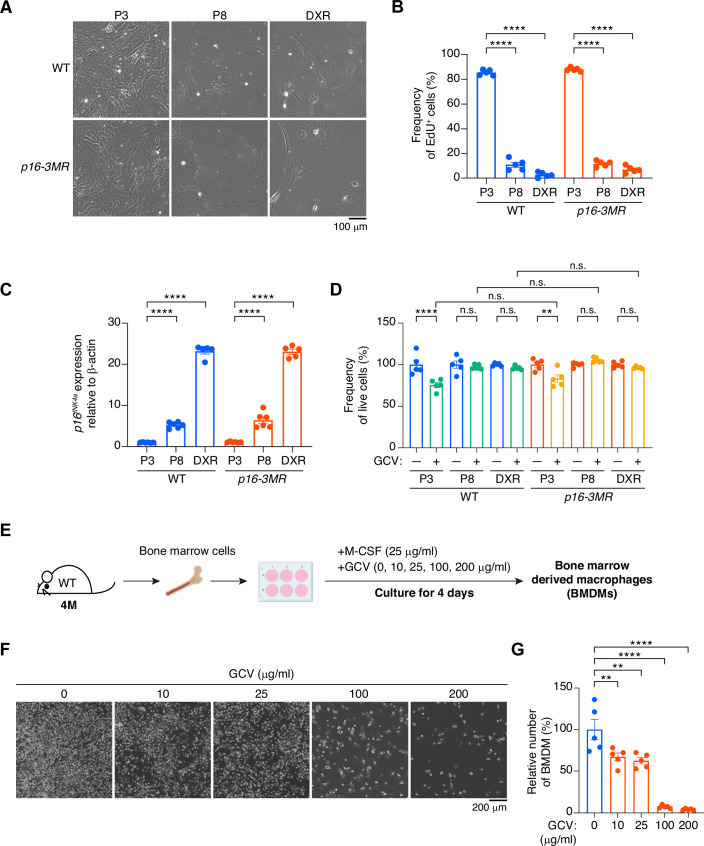
Lack of senescent cell elimination by GCV in *p16-3MR* mice in vitro. (**A**–**D**) Early-passage (P3) pre-senescent MEFs derived from WT or *p16-3MR* (MD) mouse embryos were induced to undergo cellular senescence either by serial passaging (P8) or by treatment with doxorubicin (DXR; 100 ng/mL) for 7 days. Cells were subsequently treated with DMSO (vehicle control) or ganciclovir (GCV; 10 μg/mL) for 6 days. Representative images are shown in (**A**), and EdU incorporation (**B**) and *p16*^*INK4a*^ expression (**C**) were assessed in P3, P8, or DXR-treated MEFs. Cell viability with or without GCV treatment was evaluated by MTS assay (**D**). GCV treatment, genotype, and cell condition (P3, P8, or DXR-treated) are indicated in the labels below (**D**). (**E**–**G**) Bone marrow cells were isolated from four-month-old female WT mice and differentiated into bone marrow-derived macrophages (BMDMs) using M-CSF (25 μg/mL) in the absence or presence of GCV at the indicated concentrations for 4 days (**E**). Representative images of BMDMs cultured without or with GCV at the indicated concentrations are shown in (**F**). The number of BMDMs was quantified from the images and normalized to the untreated control (**G**). The sample size (n) represents the number of technical replicates (**B**,** D**, **G**, *n* = 5; **C**, *n* = 5–6). Data were presented as mean ± s.e.m. Statistical significance was determined by one-way ANOVA followed by Dunnett’s multiple-comparison test (**B**,** C**), two-way ANOVA followed by Šídák’s multiple-comparison test (**D**), or one-way ANOVA followed by Tukey’s multiple-comparison test (**G**). **P < 0.01, ****P < 0.0001; n.s. not significant. (**B**,** C**) ****P < 0.0001 (P3 vs. P8 or DXR for both WT and *p16-3MR*). (**D**) WT: ****P < 0.0001 (P3; GCV– vs. GCV+), *P* = 0.9981 (P8; GCV– vs. GCV+), and *P* = 0.9729 (DXR; GCV– vs. GCV+). *p16-3MR*: **P = 0.0013 (P3; GCV– vs. GCV+), *P* = 0.8337 (P8; GCV– vs. GCV+), and *P* = 0.9819 (DXR; GCV– vs. GCV+). Genotype comparisons (WT vs. *p16-3MR*): *P* = 0.4084 (P3 GCV+), *P* = 0.3547 (P8 GCV+), and *P* > 0.9999 (DXR GCV+). (**G**) **P = 0.0090 (0 vs. 10 μg/ml), **P = 0.0026 (0 vs. 25 μg/ml), and ****P < 0.0001 (0 vs. 100 or 200 μg/ml). Experiments were independently repeated at least twice with similar results. Source data are available online for this figure.

**Figure EV2:**
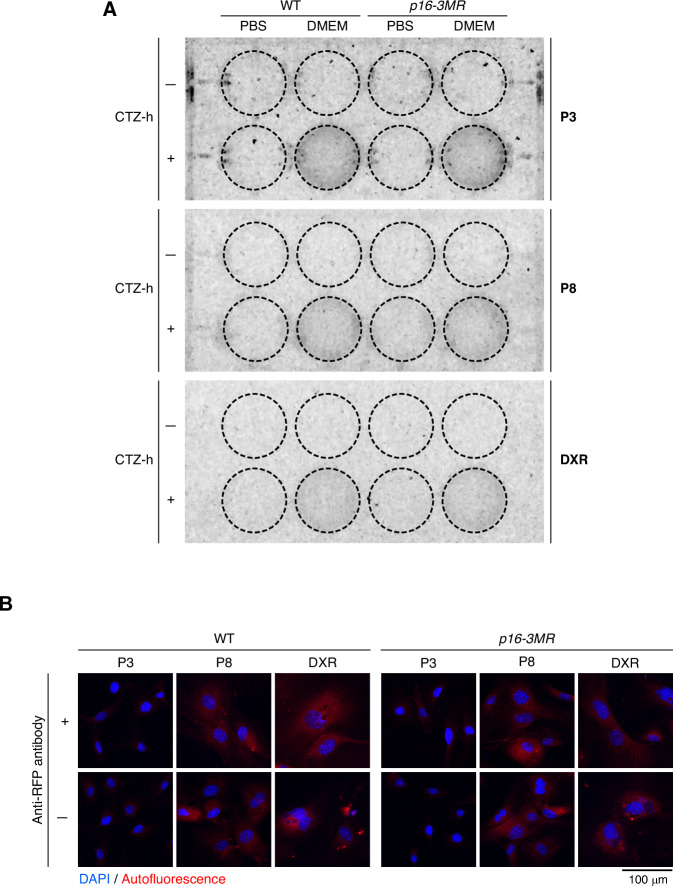
Lack of genotype-dependent luminescence and RFP signals in *p16-3MR* MEFs. (**A**) Representative bioluminescence images of early-passage (P3) and senescent (P8 or DXR-treated) mouse embryonic fibroblasts (MEFs) derived from WT or *p16-3MR* (MD) embryos. Cellular senescence was induced by serial passaging (P8) or by treatment with doxorubicin (DXR; 100 ng/mL) for 7 days. Prior to imaging, the culture medium was replaced with PBS or DMEM containing 10% FBS, in the presence or absence of CTZ-h (final concentration, 2 μg/mL). (**B**) Immunofluorescence staining of the indicated MEFs. Cells were stained with or without anti-RFP antibody. Both WT and *p16-3MR* MEFs at P8 or following DXR treatment exhibited non-specific fluorescence signals (autofluorescence) regardless of the presence of the primary antibody. Experiments were independently repeated at least twice with similar results.

Our data demonstrate that GCV administration does not alter bioluminescent signal levels in *p16-3MR* mice (Figs. 2D and 4B). These results, together with the unchanged endogenous *p16*^*INK4a*^ expression shown in Fig. EV1, strongly suggest that the *3MR* transgene does not exhibit detectable functional activity in *p16-3MR* mice. To further assess the functionality of the *3MR* transgene, we performed side-by-side analyses using cultured mouse embryonic fibroblasts (MEFs) derived from *p16-3MR* mice and WT littermates. Early-passage (P3) non-senescent MEFs as well as late-passage (P8) or doxorubicin-induced senescent MEFs were examined under controlled in vitro conditions (Fig. 5A–C). Neither senescent *p16-3MR* MEFs nor WT MEFs exhibited genotype-dependent increases in bioluminescence signals, as judged by BLI (Fig. EV2A). Notably, luminescence slightly increased when CTZ-h was added to serum-containing culture medium (DMEM), but not when added to PBS (Fig. EV2A), consistent with serum albumin–dependent CTZ luminescence reported previously (Zhao et al, 2004). Because the *3MR* construct also includes an RFP reporter, we next evaluated RFP fluorescence as an independent readout of transgene activity. RFP fluorescence imaging revealed only weak signals in senescent MEFs from both *p16-3MR* and WT mice, irrespective of anti-RFP antibody staining (Fig. EV2B). These findings are consistent with, and may reflect, well-known senescence-associated cellular autofluorescence (Bertolo et al, 2019). Furthermore, GCV treatment did not significantly reduce the number of senescent cells in *p16-3MR* MEFs compared with WT MEFs under identical conditions (Fig. 5D). Collectively, these results indicate that, at least under our experimental conditions, the *3MR* transgene is not expressed at levels sufficient to confer detectable reporter activity or functional GCV sensitivity.

It should also be noted that GCV has been reported to inhibit the proliferation of macrophages, microglia, and T cells in a TK-independent manner and to suppress activation of the cGAS/STING pathway (Ding et al, 2014; Battiwalla et al, 2007; Gong et al, 2022). Several immune cell populations, including macrophages, microglia, and subsets of T cells, are known to exhibit relatively high levels of *p16*^*INK4a*^ expression (Hall et al, 2016, 2017; Matsudaira et al, 2023; Tufa et al, 2025), and the cGAS/STING pathway itself has been implicated in the induction of cellular senescence and the SASP (Dou et al, 2017; Glück et al, 2017; Takahashi et al, 2018; Yang et al, 2017). Thus, GCV administration may influence immune cell populations and/or modulate cGAS/STING signaling independently of HSV-TK activity, rather than selectively eliminating *p16*^*INK4a*^-expressing senescent cells in the *p16-3MR* model. Consistent with this possibility, we observed that GCV reduced the number of macrophages derived from WT mouse bone marrow (Fig. 5E–G). Collectively, these findings indicate that phenotypic changes observed following GCV treatment in *p16-3MR* mice—such as reduced *p16*^*INK4a*^ expression or attenuated inflammatory responses—cannot be unequivocally attributed to the selective elimination of senescent cells via transgene-encoded TK activity. These results underscore the necessity of including appropriate negative controls, such as WT mice analyzed in parallel, when interpreting GCV-mediated effects in this model.

**Figure EV3:**
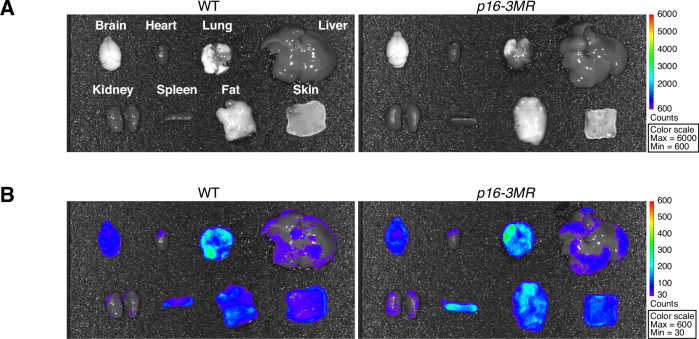
No significant difference in ex vivo bioluminescence in organs between *p16-3MR* and WT mice. (**A**,** B**) Organs were harvested from 5-month-old WT and *p16-3MR* (JC) mice and incubated in CTZ-h diluted 1:10 in PBS (final concentration, 15 μg/mL) for 45 min according to the protocol described by Wang et al, (2026). Ex vivo bioluminescence imaging was performed using an IVIS imaging system. The same imaging data are displayed using different color scale ranges: minimum and maximum thresholds were set to 600 and 6,000 counts in (**A**) and to 30 and 600 counts in (**B**). The binning setting was “medium” for all acquisitions. Color bars represent signal intensity (counts) corresponding to the indicated minimum and maximum thresholds. Experiments were independently repeated at least twice with similar results.

Our study was unable to reproduce the reported functionality of the *p16-3MR* mouse model described by Demaria et al, (2014). However, because multiple studies using *p16-3MR* mice have subsequently been published (Baar et al, 2017; Chang et al, 2016; Demaria et al, 2017; Jeon et al, 2017; Kaur et al, 2023; Moiseeva et al, 2023), it remains possible that the *3MR* transgene retains partial functionality in specific biological contexts. To further explore this possibility, we evaluated a recently reported approach involving incubation of dissected organs from *p16-3MR* mice in diluted CTZ-h solution for 45–60 min (Wang et al, 2026). Under these conditions, bioluminescent signals were detectable only at low detection thresholds and were comparable between *p16-3MR* and WT tissues (Fig. EV3), indicating that the observed signals are most consistent with substrate-dependent background rather than Rluc activity. Collectively, we did not identify experimental conditions in which the *p16-3MR* model reliably reflected endogenous *p16*^*INK4a*^ expression. While we cannot formally exclude the possibility that the *p16-3MR* mouse model may retain some functionality under certain circumstances, our findings strongly suggest that data obtained at detection thresholds set substantially below the level recommended by the IVIS system (Revvity Inc.) and/or in the absence of WT controls should be interpreted with caution.

Finally, we considered why the limitations of the *p16-3MR* mouse model remained unrecognized for more than a decade after its initial description. A major contributing factor may have been the underappreciation of an important property of CTZ-h: even in the absence of Rluc, coelenterazine can generate luminescent signals through reactions with serum albumin (Zhao et al, 2004) and superoxide (Bronsart et al, 2016). Although the fact that CTZ-h reacts with serum albumin to produce luminescence had been reported prior to the publication of the original *p16-3MR* study, its potential impact on CTZ-h–based in vivo BLI does not appear to have been fully considered. In addition, the existence of multiple published studies employing *p16-3MR* mice may have further obscured these limitations by reinforcing confidence in the model’s performance. Given the well-recognized publication bias favoring positive findings, negative observations may have remained largely unreported, thereby contributing to an overestimation of the model’s reliability (Nat Hum Behav, 2019).

Taken together, our findings demonstrate that bioluminescence signals detected in the *p16-3MR* model are most consistent with substrate-dependent background under the conditions tested, and do not reliably reflect endogenous *p16*^*INK4a*^ expression. These results underscore the need for careful interpretation of studies using the *p16-3MR* mode, particularly in aging and senolytics research.

## Methods

**Table:** Reagents and tools table

Reagent/resource	Reference or source	Identifier or catalog number
**Experimental models**		
C57BL/6J (*M. musculus*)	CLEA Japan, the National BioResource Project of the Ministry of Education, Culture, Sports, Science and Technology in Japan	C57BL/6JJcl
C57BL/6J (*M. musculus*)	Jackson Laboratory Japan	C57BL/6J
Albino C57BL/6N (*M. musculus*)	Jackson Laboratory Japan	B6N-Tyrc-Brd/BrdCrCrl
*p16-3MR* mice (*M. musculus*)	Demaria et al, 2014	
Albino *p16-luc* mice (*M. musculus*)	Kawamoto et al, 2023	
**Antibodies**		
Rat anti-RFP antibody	ChromoTek	5F8
Alexa Fluor Plus 555 donkey anti-rat IgG	Invitrogen	A48270
**Oligonucleotides and other sequence-based reagents**		
Mouse *Actb* primers	Kawamoto et al, 2023	Methods
Mouse *p16*^*INK4a*^ primers	Kawamoto et al, 2023	Methods
**Chemicals, Enzymes and other reagents**		
Ganciclovir	G2536-100MG	
Doxorubicin Hydrochloride	FUJIFILM Wako Pure Chemical	046-21523
IVISbrite CTZ-h, RediJect Solution	Perkin Elmerr/Revvity	760506
Coelenterazine-h	FUJIFILM Wako Pure Chemical Corporation	035-22991
*D*-luciferin substrate	FUJIFILM Wako Pure Chemical Corporation	126-05116
DMEM	Nacalai tesque	08458-16
Fetal bovine serum	Sigma-Aldrich	173012
Penicillin–streptomycin	Sigma-Aldrich	P4333-100ML
2.5 g/l-Trypsin Solution	Nacalai Tesque	35555-54
Click-iT EdU Cell Proliferation Kit for Imaging	Invitrogen	C10640
Dimethyl sulfoxide	Nacalai tesque	13445-74
CellTiter 96 AQueous One Solution Cell Proliferation Assay	Promega	G3582
Polyoxyethylene Sorbitan Monolaurate (Tween 20)	Nacalai Tesque	28353-85
DAPI	Dojindo	D523
TrueBlack Lipofuscin Autofluorescence Quencher	Biotium	23007
Fluoromount-G	SouthernBiotech	0100-01
TRIzol	Thermo Fisher	15596018
RNeasy Mini Kit	Qiagen	74106
PrimeScript RT Reagent Kit with gDNA Eraser	Takara Bio	RR047A
TB Green Premix Ex Taq II	Takara Bio	RR820A
RBC Lysis Buffer	BioLegend	420301
M-CSF	FUJIFILM Wako Pure Chemical Corporation	135-14391
**Software**		
Living Image Software v 4.7.3	PerkinElmer/Revvity	
Prism v10.5.0	GraphPad software	
**Other**		
IVIS Lumina Series III	PerkinElmer/Revvity Inc.	
ImageQuant 800 system	Cytiva	
Thermal Cycler Dice Real Time System III	Takara Bio	
All-in-One Fluorescence Microscope	Keyence	BZ-X810
Electric shaver	Panasonic	ER803P
6-mm biopsy punch	Kai Industries	BP-60F
70-µm cell strainer	Greiner	542070

All of the animal experiments were approved by the Animal Research Committee of the Research Institute for Microbial Diseases (RIMD), The University of Osaka (Biken-AP-R07-05-0).

### Mice

Wild-type (WT) C57BL/6 mice were purchased from the Jackson Laboratory Japan, Inc. and CLEA Japan. Some aged WT mice were provided by the Foundation for Biomedical Research and Innovation at Kobe through the National BioResource Project of the Ministry of Education, Culture, Sports, Science and Technology in Japan. The *p16-3MR* mice (Demaria et al, 2014) were initially provided by Dr. Judith Campisi (Buck Institute for Research on Aging, USA) via Dr. Charles Fouillade (Institute Curie Research Center, France) in 2016, and subsequently by Dr. Marco Demaria (European Research Institute for the Biology of Ageing, Netherlands) in 2023. Albino *p16-3MR* mice were generated by crossing with C57BL/6 albino mice (Jackson Laboratory Japan, Inc.) for six generations. Albino *p16-luc* mice were generated by crossing with C57BL/6 albino mice (Charles River Laboratories Japan) for six generations (Yamakoshi et al, 2009; Kawamoto et al, 2023). Mice were housed at 23 ± 2 °C, 55 ± 15% humidity, under a 12-h light/12-h dark cycle, and fed a normal diet sterilized by 20 kGy gamma irradiation (CE-2; CLEA Japan Inc.). For ganciclovir (GCV; G2536-100MG, Sigma-Aldrich) treatment, mice received daily intraperitoneal (i.p.) injections of 25 mg/kg GCV in PBS for five consecutive days. Control mice received the same volume of PBS. For doxorubicin (DXR; 046-21523, FUJIFILM Wako Pure Chemical) treatment, mice received a single intraperitoneal injection of 10 mg/kg DXR in PBS. Control mice received the same volume of PBS. Mice were euthanized using carbon dioxide, and all efforts were made to minimize suffering. To minimize potential confounding factors, mice that exhibited tumor development were excluded from all downstream analyses.

### Bioluminescence imaging

All imaging experiments were performed using an IVIS Lumina Series III system (PerkinElmer/Revvity Inc.). Imaging parameters were as follows: exposure time, 5 min; binning, medium or large as indicated in the figure legends. For non-invasive bioluminescence imaging (BLI) of *p16-3MR* mice, animals received intraperitoneal (i.p.) injections of 15 µg IVISbrite Coelenterazine-h RediJect Solution (760506; PerkinElmer/Revvity). Twenty-five minutes later, mice were anesthetized with isoflurane, and luminescence signals were acquired for 5 min. Hair was removed prior to BLI using an electric shaver (ER803P; Panasonic). For wound-healing experiments, a full-thickness excisional wound was generated at the center of the dorsal skin using a 6-mm biopsy punch (BP-60F; Kai Industries). Ganciclovir (GCV) or PBS was administered once daily on days 1–5 post-wounding, and BLI was performed on the indicated days. For ex vivo organ BLI, after the IVISbrite Coelenterazine-h RediJect Solution became unavailable, an alternative Coelenterazine-h (CTZ-h) solution was prepared according to the manufacturer’s instructions. Briefly, CTZ-h (035-22991, FUJIFILM Wako Pure Chemical) was dissolved in propylene glycol at 0.25 mg/mL and mixed with 50 mM citrate buffer at a 6:4 (v/v) ratio to yield a final concentration of 150 μg/mL. Ex vivo imaging was performed according to a previously reported protocol (Wang et al, 2026), with harvested tissues incubated for 45–60 min in CTZ-h diluted 1:10 in PBS prior to imaging. For non-invasive BLI of *p16-luc* mice, animals were anesthetized with isoflurane and injected i.p. with D-luciferin (75 mg/kg; 126-05116; FUJIFILM Wako Pure Chemical Corporation; 30 mg/mL in saline). Imaging parameters were as follows: exposure time, 5 min; binning, medium. Imaging data were analyzed using Living Image software (version 4.7.3; PerkinElmer/Revvity Inc.). Although luminescence measurements are typically reported in radiance, data are presented here in counts to maintain consistency with Demaria et al, (2014). Investigators were not blinded to group allocation during experiments or outcome assessment.

### Whole-genome sequencing

Long-read sequencing: The extracted gDNA solution was so viscous that it was forming clear threads while being aspirated by a pipette, and had a clump of white threads floating. The gDNA was purified before being submitted to the Nanopore library prep. About 50 μL of the gDNA solution was diluted by eightfold, adding 350 μL of Buffer EB. Then it was incubated in the Eppendorf ThermoMixer C for 2 h at 65 °C without agitation. After the incubation, the viscosity of the solution was reduced, and although a clump of white threads was still present, it no longer formed clear threads while being aspirated. The solution was further purified using AMPure XP in a 1.0x ratio. The gDNA was eluted with 200 μL of Buffer EB. The DNA concentration of the purified solution was quantified using Qubit. To adjust the concentration and the volume of the solution adequate for Nanopore library prep, it was concentrated 4-fold using AMPure XP in a 1.0x ratio and eluted with 50 μL of Buffer EB. 5 μg of the concentrated purified gDNA was used to make a Nanopore library by the ligation method without fragmentation using SQK-LSK109. Then the library was submitted to the PromethION Flow Cell (R9.4.1) and analyzed by the P2 solo. The basecalling was conducted by Guppy version 6.5.7, 450 bps super-accurate mode. Short-read sequencing: The illumina libraries were prepared using Illumina DNA PCR-Free Prep and sequenced on an Illumina NovaSeq X Plus with 150 bp paired-end mode. Sequence reads were mapped onto the custom genome with the mouse reference genome (mm10), pTARBAC, and *3MR* sequences.

### Establishment and culture of mouse embryonic fibroblasts (MEFs)

MEFs were isolated from E13.5–15.5 embryos of pregnant C57BL/6 WT or *p16-3MR* mice. Pregnant mice were euthanized, and embryos, including the amniotic sac, were collected into PBS containing 1% penicillin–streptomycin (P/S) (P4333-100ML; Sigma-Aldrich). Heads, tails, limbs, and internal organs were removed, and the remaining tissue was transferred to a biosafety cabinet. The tissue was minced with sterilized scissors, suspended in 1 mL PBS (1% P/S), and digested with 2 mL of 0.25% trypsin solution (35555-54; Nacalai Tesque) at 37 °C with gentle shaking (150 rpm) for 20 min. Digestion was terminated by adding Dulbecco’s Modified Eagle Medium (DMEM; 08458-16; Nacalai Tesque) supplemented with 10% fetal bovine serum (FBS; 173012; Sigma-Aldrich) and 1% P/S. Cell clumps were dissociated by pipetting and vortexing, then centrifuged at 1500 rpm for 5 min at 4 °C. The resulting pellet was resuspended and cultured in DMEM supplemented with 10% FBS and 1% P/S. Early-passage MEFs (P3) were used as controls, and late-passage MEFs (P8) that had ceased proliferation were used as replicative senescent cells. For induction of doxorubicin (DXR)-induced senescence, MEFs were treated with 100 ng/mL DXR for 7 days, followed by medium replacement with fresh DMEM supplemented with 10% FBS and 1% P/S to confirm growth arrest. Experiments were performed 3 days after medium replacement. For proliferation assays, MEFs were incubated with 2 µM 5-ethynyl-2′-deoxyuridine (EdU) for 3 days, fixed with 4% paraformaldehyde (PFA), and stained using the Click-iT EdU Cell Proliferation Kit for Imaging (C10640; Invitrogen). For cell viability assays, MEFs were treated with 10 µg/mL GCV or vehicle (Dimethyl sulfoxide (DMSO; 13445-74, Nacalai tesque)) for 6 days, with medium replaced every 2 days. Cell viability was assessed using the CellTiter 96 AQueous One Solution Cell Proliferation Assay (MTS; G3582; Promega), and GCV-treated MEFs were normalized to untreated controls. For RFP staining, MEFs cultured on glass slides were fixed with 4% PFA for 20 min at room temperature and washed with PBS. Cells were permeabilized with PBS containing 0.05% Tween 20 (28353-85; Nacalai Tesque) and blocked with 2.5% donkey serum. Cells were then incubated with an anti-RFP antibody (5F8; ChromoTek; 1:400), followed by Alexa Fluor Plus 555 donkey anti-rat IgG (A48270; Invitrogen; 1:1000) as the secondary antibody. Cells were stained with DAPI (D523; Dojindo) and treated with TrueBlack Lipofuscin Autofluorescence Quencher (23007; Biotium) before mounting with Fluoromount-G (0100-01; SouthernBiotech). For BLI of MEFs, cells were seeded in 12-well plates and the medium was replaced with PBS, or DMEM containing 10% FBS, each supplemented with CTZ-h (final concentration; 2 μg/mL) or left unsupplemented as a control. Luminescence images were acquired using an ImageQuant 800 system (Cytiva).

### Quantitative real-time PCR analysis

Cultured MEFs and tissues were lysed in TRIzol reagent (15596018; Thermo Fisher Scientific), and total RNA was purified using the RNeasy Mini Kit (74106; Qiagen) according to the manufacturer’s instructions. Genomic DNA was removed, and cDNA was synthesized using the PrimeScript RT Reagent Kit with gDNA Eraser (RR047A; Takara Bio). RT-qPCR was performed using TB Green Premix Ex Taq II (RR820A; Takara Bio) on a Thermal Cycler Dice Real Time System III (Takara Bio). *p16*^*INK4a*^ expression levels were normalized to *Actb*. The primer sequences were as follows:

*Actb*: forward, GATGACCCAGATCATGTTTGA; reverse, GGAGAGCATAGCCCTCGTAG

*p16*^*INK4a*^: forward, GAACTCTTTCGGTCGTACCC; reverse, CGAATCTGCACCGTAGTTGA

### Bone marrow-derived macrophage (BMDM) experiments

BMDMs were prepared as previously described (Toda et al, 2020) with minor modifications. Briefly, 4-month-old C57BL/6 female mice were euthanized in accordance with an institutionally approved protocol, and femurs and tibias were collected. Excess muscle tissue and epiphyses were removed, and bone marrow was flushed out with DMEM supplemented with 10% FBS using a 23-gauge needle. The cell suspension was passed through a 70-µm cell strainer (542070; Greiner) and centrifuged at 1500 rpm for 10 min at 4 °C. The pellet was resuspended in 1 mL 1× RBC Lysis Buffer (420301; BioLegend) and incubated on ice for 1 min to lyse red blood cells. Bone marrow cells were then cultured in DMEM containing 10% FBS and 1% P/S and differentiated into BMDMs in the presence of macrophage colony-stimulating factor (M-CSF; 25 ng/mL; 135-14391; FUJIFILM Wako Pure Chemical Corporation) for 4 days. During differentiation, GCV dissolved in DMSO was added at final concentrations of 0, 10, 25, 100, or 200 µg/mL. BMDMs cultured on glass slides were stained with DAPI (D523; Dojindo) for 10 min at room temperature and mounted using Fluoromount-G (0100-01; SouthernBiotech). Images were randomly acquired using a BZ-X810 fluorescence microscope (Keyence). Cell numbers were counted from the images and normalized to those of the untreated control group. The investigators were not blinded to allocation during experiments and outcome assessment.

### Statistics

The graphs or plots are presented as mean ± s.e.m. (standard error of the mean) with *n* ≥ 3 as indicated in the figure legends. Data were assumed to follow a normal distribution, but this was not formally tested. All data were visualized and analyzed using GraphPad Prism (version 10.5.0). Statistical significance was assessed by two-way ANOVA followed by Tukey’s multiple comparisons test (Fig. 2B,F), one-way ANOVA followed by Tukey’s multiple comparisons test (Figs. 3C,E and 5G), two-way ANOVA followed by Šídák’s multiple comparisons test (Figs. 4C,F and 5D), one-way ANOVA followed by Dunnett’s multiple comparisons test (Fig. 5B,C) or one-way ANOVA followed by Šídák’s multiple comparisons test (Fig. EV1A–F). *P* < 0.05 was considered statistically significant. Significance levels were denoted as follows: *P < 0.05; **P < 0.01; ****P < 0.0001; n.s. not significant. Sample sizes were determined based on previous experience with similar experiments in our laboratory.

## Supplementary information

Peer Review File

Source data Fig. 2

Source data Fig. 3

Source data Fig. 4

Source data Fig. 5

Expanded View Figures

## Data Availability

This study includes no data deposited in external repositories. Source data underlying Figs. 2B,F, 3C,E, 4C,4F, and 5A–D, F,G are provided as Source Data files. The source data of this paper are collected in the following database record: biostudies:S-SCDT-10_1038-S44319-026-00802-8.
