# Supplementary figures and images for: Limitations of the p16-3MR mouse model for detecting and eliminating senescent cells
Source: EMBO Rep. 2026 May 28;27(13):3547–63. doi: 10.1038/s44319-026-00802-8 (PMC13354569; doi:10.1038/s44319-026-00802-8)

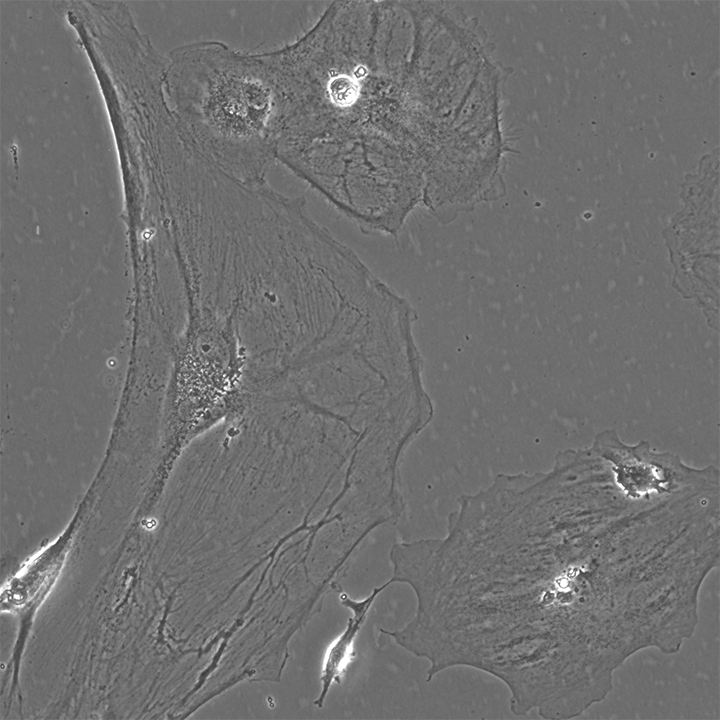

Supplement: Supplementary file 5 — Source data Fig. 5 [file 44319_2026_802_MOESM5_ESM.zip › Figure 5/5A/5A_p163MR_DXR.tif]

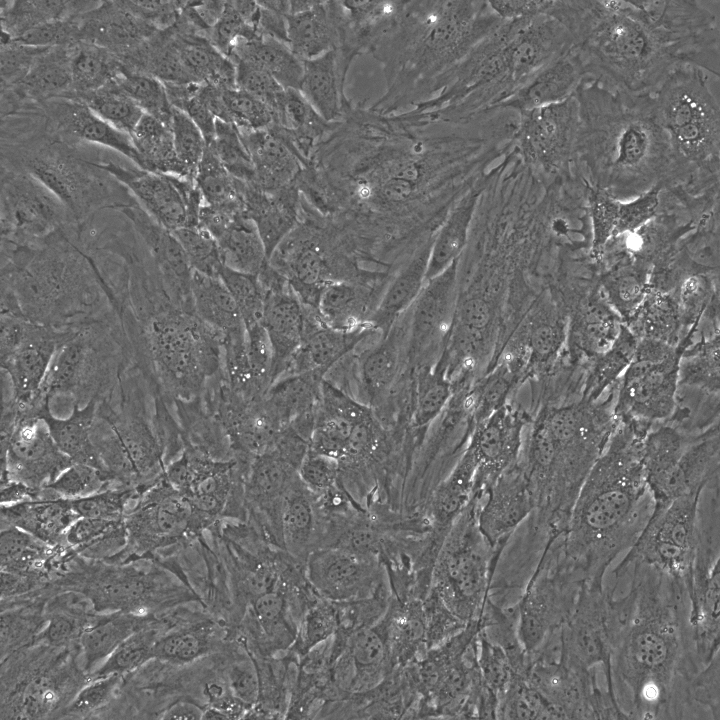

Supplement: Supplementary file 5 — Source data Fig. 5 [file 44319_2026_802_MOESM5_ESM.zip › Figure 5/5A/5A_p163MR_P3.tif]

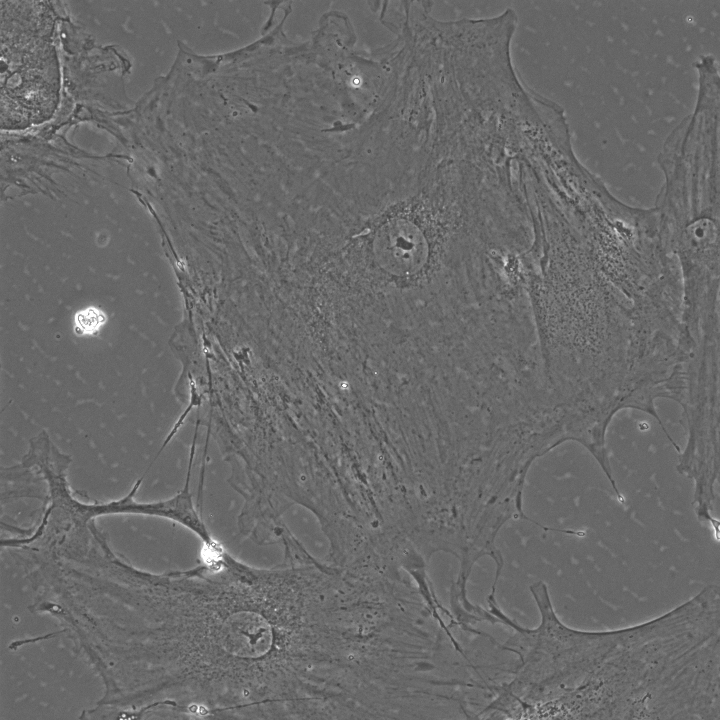

Supplement: Supplementary file 5 — Source data Fig. 5 [file 44319_2026_802_MOESM5_ESM.zip › Figure 5/5A/5A_p163MR_P8.tif]

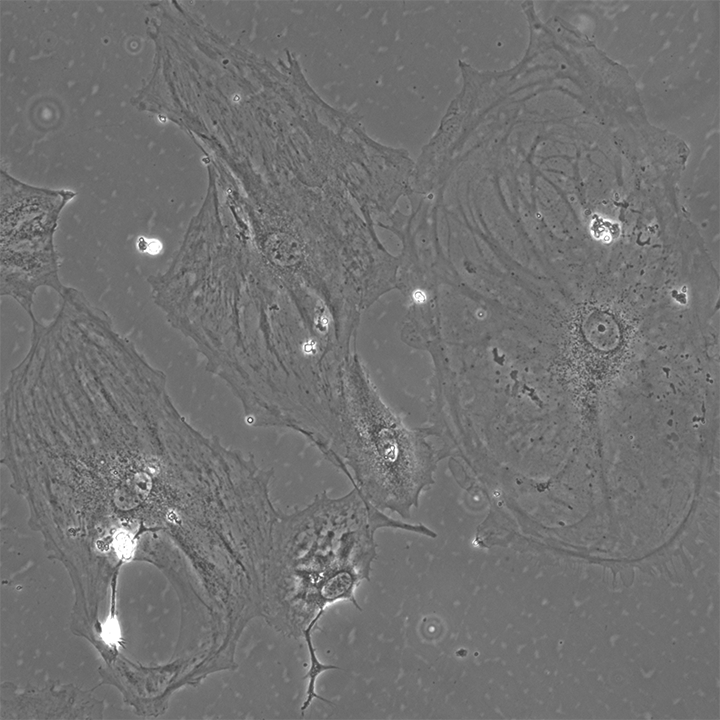

Supplement: Supplementary file 5 — Source data Fig. 5 [file 44319_2026_802_MOESM5_ESM.zip › Figure 5/5A/5A_WT_DXR.tif]

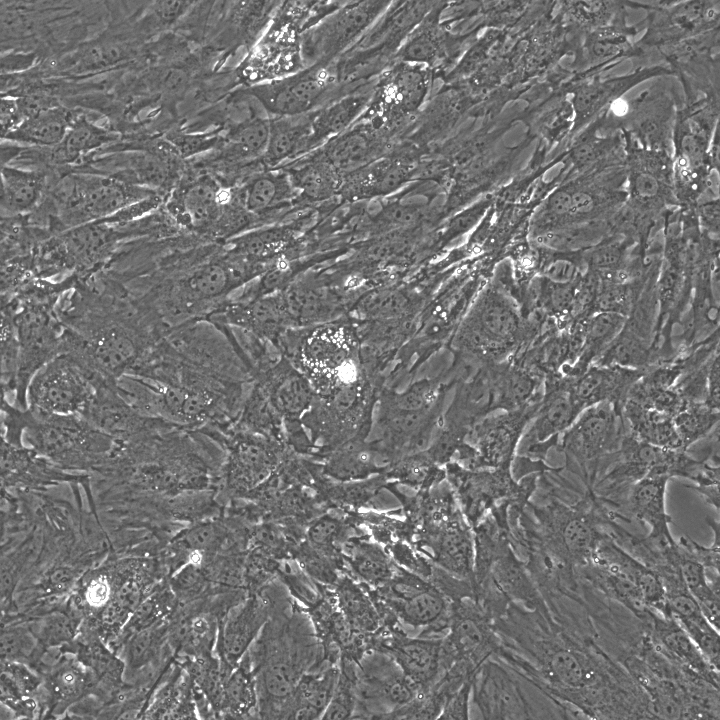

Supplement: Supplementary file 5 — Source data Fig. 5 [file 44319_2026_802_MOESM5_ESM.zip › Figure 5/5A/5A_WT_P3.tif]

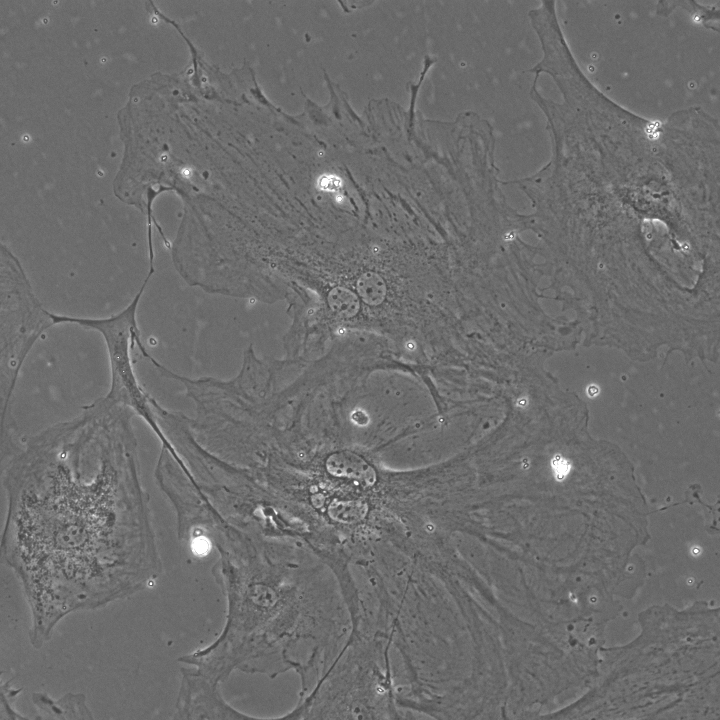

Supplement: Supplementary file 5 — Source data Fig. 5 [file 44319_2026_802_MOESM5_ESM.zip › Figure 5/5A/5A_WT_P8.tif]

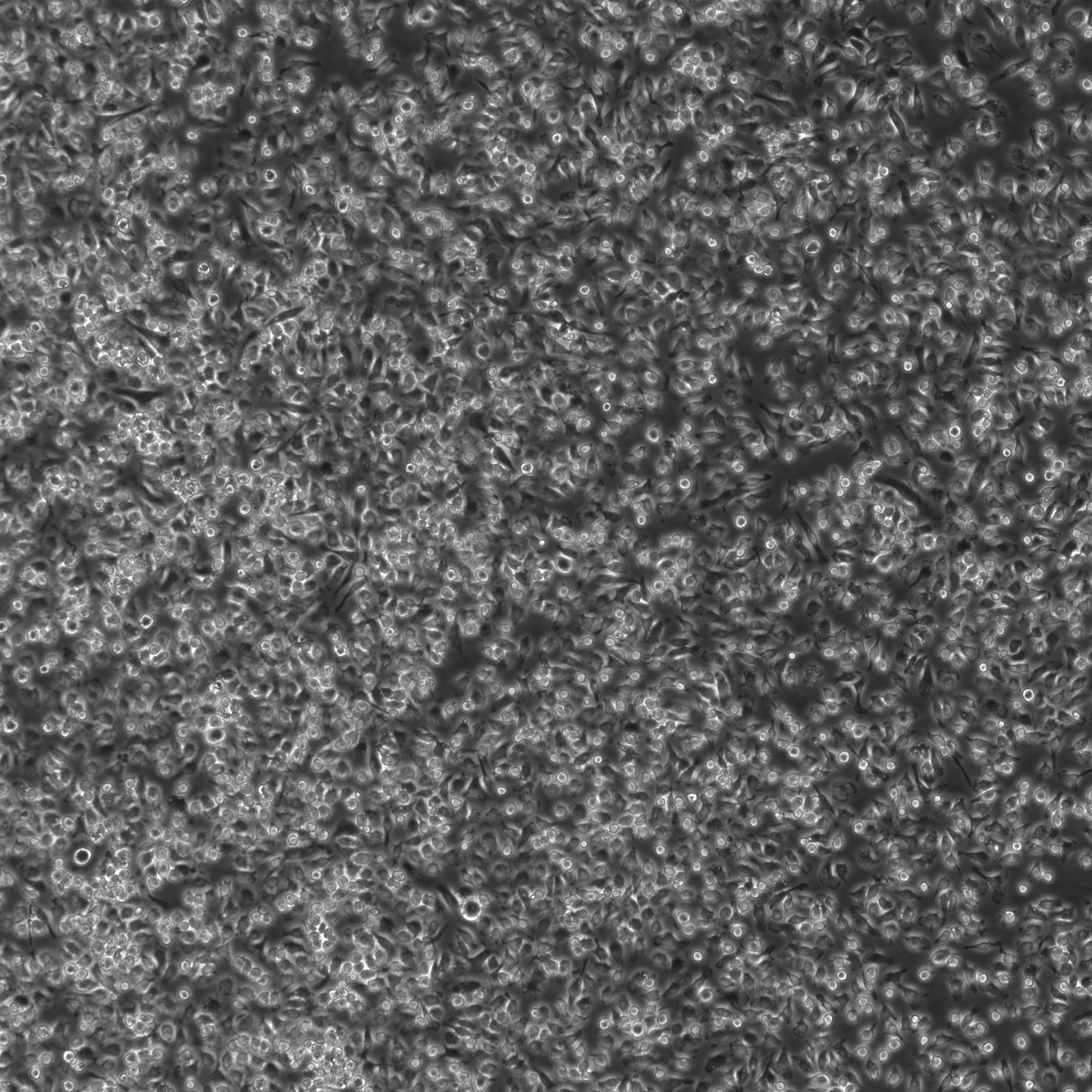

Supplement: Supplementary file 5 — Source data Fig. 5 [file 44319_2026_802_MOESM5_ESM.zip › Figure 5/5F/5F_BMDM_GCV0.tif]

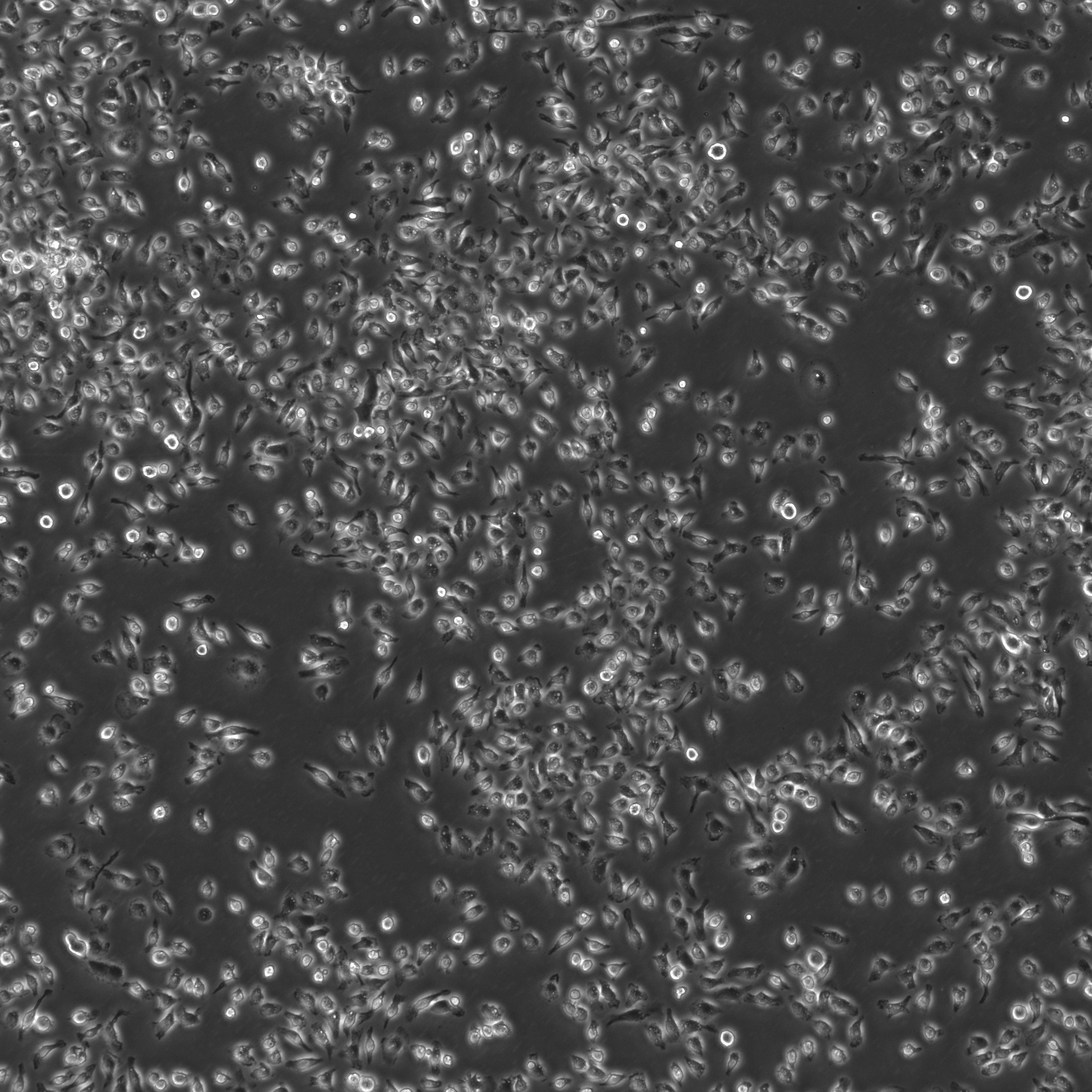

Supplement: Supplementary file 5 — Source data Fig. 5 [file 44319_2026_802_MOESM5_ESM.zip › Figure 5/5F/5F_BMDM_GCV10.tif]

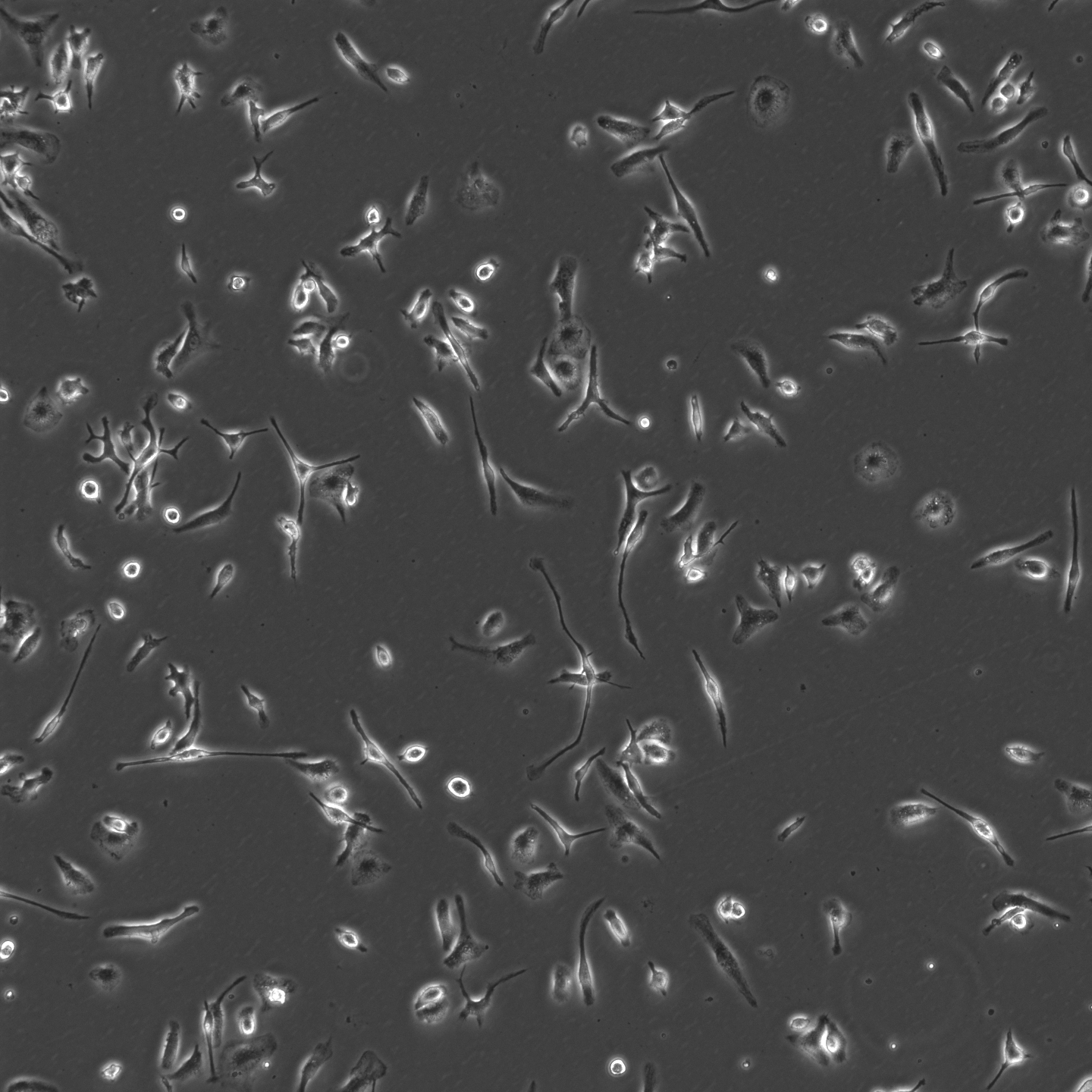

Supplement: Supplementary file 5 — Source data Fig. 5 [file 44319_2026_802_MOESM5_ESM.zip › Figure 5/5F/5F_BMDM_GCV100.tif]

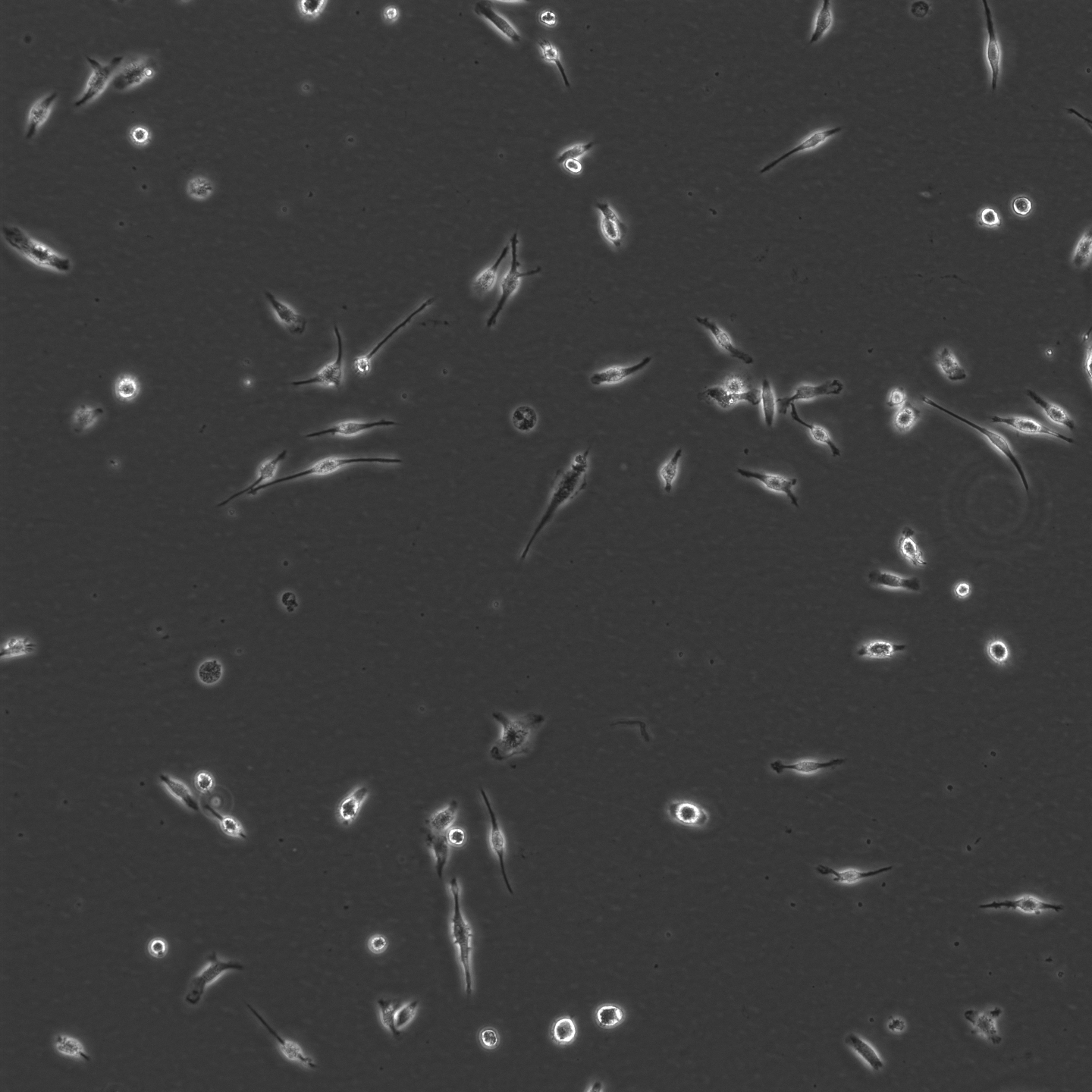

Supplement: Supplementary file 5 — Source data Fig. 5 [file 44319_2026_802_MOESM5_ESM.zip › Figure 5/5F/5F_BMDM_GCV200.tif]

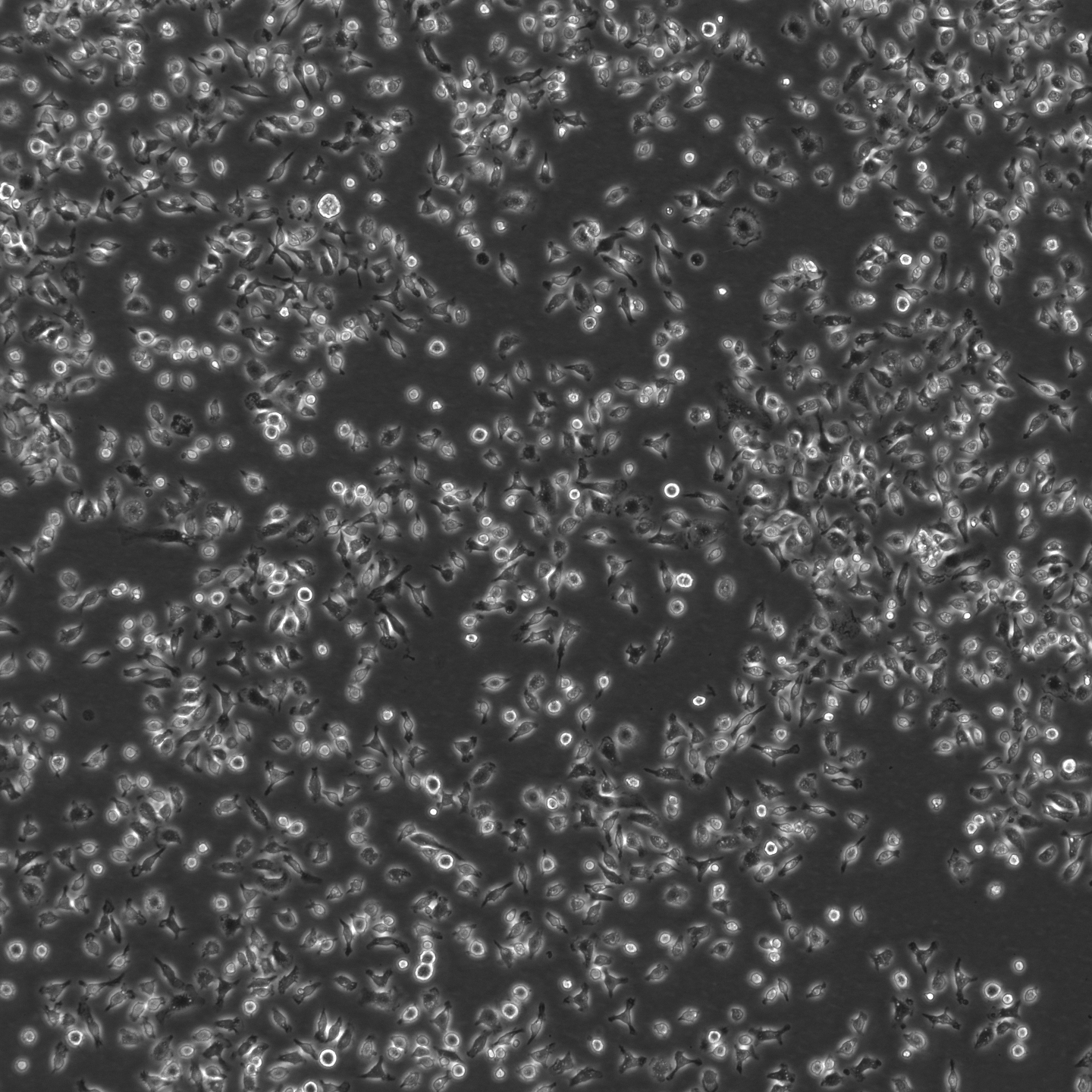

Supplement: Supplementary file 5 — Source data Fig. 5 [file 44319_2026_802_MOESM5_ESM.zip › Figure 5/5F/5F_BMDM_GCV25.tif]
