# Supplementary material for: Limitations of the p16-3MR mouse model for detecting and eliminating senescent cells
Source: EMBO Rep. 2026 May 28;27(13):3547–63. doi: 10.1038/s44319-026-00802-8 (PMC13354569; doi:10.1038/s44319-026-00802-8)
Supplement: Supplementary file 6 — Expanded View Figures [file 44319_2026_802_MOESM6_ESM.pdf]

Expanded View Figures

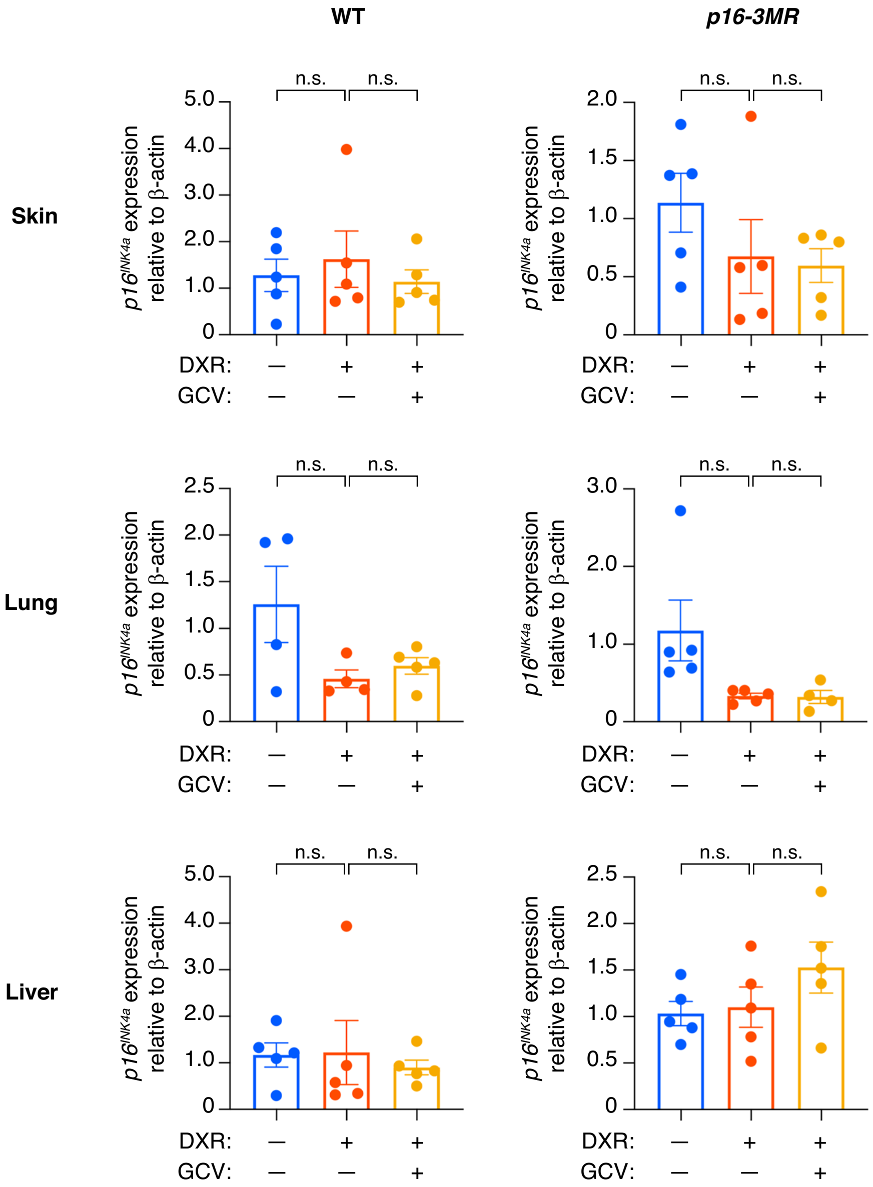

Figure EV1. No change in $p16^{INK4a}$ expression in *p16-3MR* tissues after DXR or GCV treatment.

Three-month-old female WT and *p16-3MR* (MD) mice (the same cohort used in Fig. 4A–C) were intraperitoneally injected with PBS (vehicle control) or doxorubicin (DXR; 10 mg/kg) on day 0. From days 5 to 9, mice received daily intraperitoneal injections of PBS (vehicle control) or ganciclovir (GCV; 25 mg/kg). On day 10, $p16^{INK4a}$ expression levels in the skin, lung, and liver were analyzed by RT-qPCR. Data were presented as relative $p16^{INK4a}$ expression normalized to the untreated control group (DXR−/GCV−). The sample size (n) represents the number of biological replicates ($n = 4$–5). Data were presented as mean ± s.e.m. Statistical significance was determined by one-way ANOVA followed by Šídák's multiple-comparison test. n.s. not significant. Experiments were independently repeated at least twice with similar results.

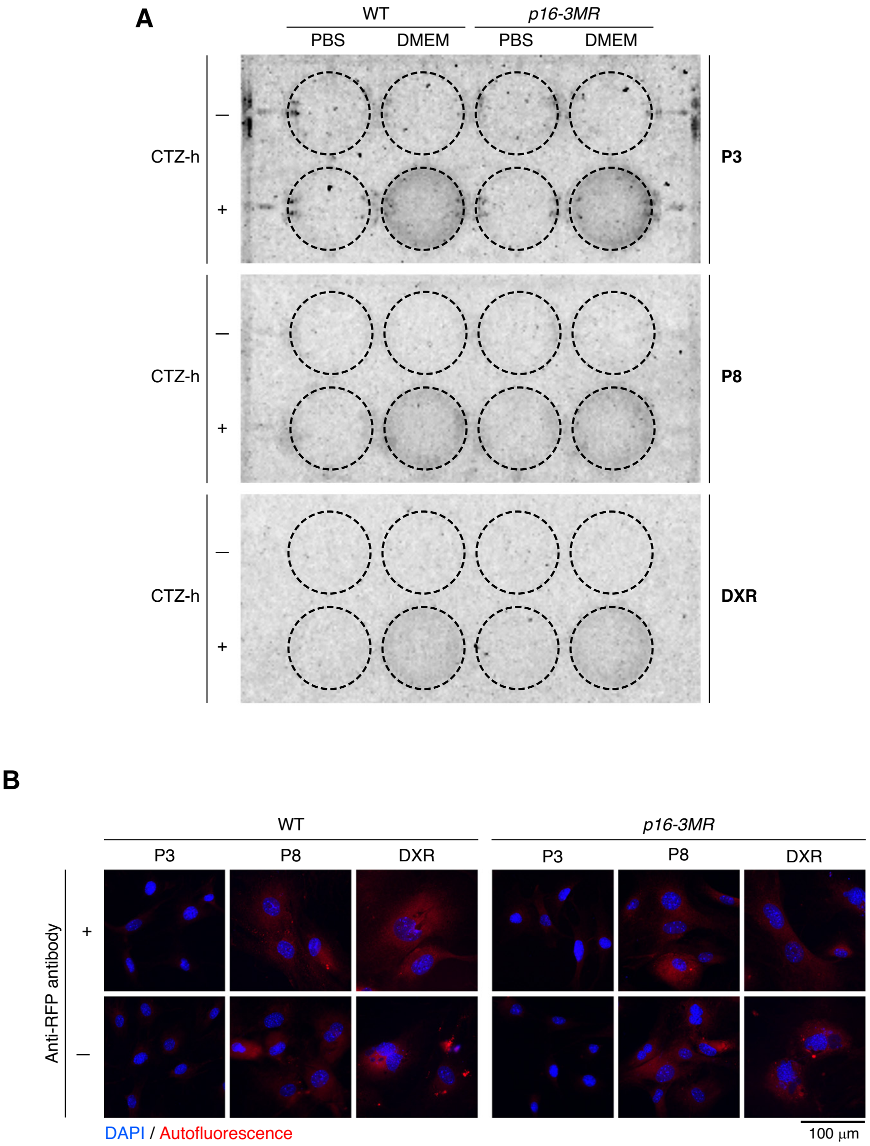

Figure EV2. Lack of genotype-dependent luminescence and RFP signals in *p16-3MR* MEFs.

(**A**) Representative bioluminescence images of early-passage (P3) and senescent (P8 or DXR-treated) mouse embryonic fibroblasts (MEFs) derived from WT or *p16-3MR* (MD) embryos. Cellular senescence was induced by serial passaging (P8) or by treatment with doxorubicin (DXR; 100 ng/mL) for 7 days. Prior to imaging, the culture medium was replaced with PBS or DMEM containing 10% FBS, in the presence or absence of CTZ-h (final concentration, 2 µg/mL). (**B**) Immunofluorescence staining of the indicated MEFs. Cells were stained with or without anti-RFP antibody. Both WT and *p16-3MR* MEFs at P8 or following DXR treatment exhibited non-specific fluorescence signals (autofluorescence) regardless of the presence of the primary antibody. Experiments were independently repeated at least twice with similar results.

 © The Author(s)

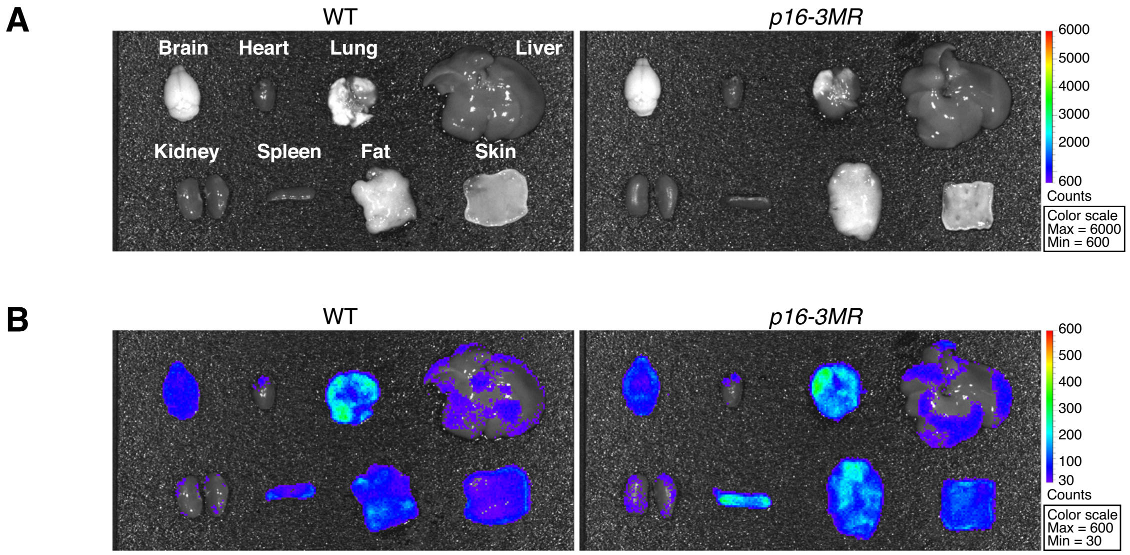

Figure EV3. No significant difference in ex vivo bioluminescence in organs between *p16-3MR* and WT mice.

(**A**, **B**) Organs were harvested from 5-month-old WT and *p16-3MR* (JC) mice and incubated in CTZ-h diluted 1:10 in PBS (final concentration, 15 μg/mL) for 45 min according to the protocol described by Wang et al, (2026). Ex vivo bioluminescence imaging was performed using an IVIS imaging system. The same imaging data are displayed using different color scale ranges: minimum and maximum thresholds were set to 600 and 6,000 counts in (**A**) and to 30 and 600 counts in (**B**). The binning setting was “medium” for all acquisitions. Color bars represent signal intensity (counts) corresponding to the indicated minimum and maximum thresholds. Experiments were independently repeated at least twice with similar results.

© The Author(s)
