# Supplementary material for: Limitations of the p16-3MR mouse model for detecting and eliminating senescent cells
Source: EMBO Rep. 2026 May 28;27(13):3547–63. doi: 10.1038/s44319-026-00802-8 (PMC13354569; doi:10.1038/s44319-026-00802-8)
Supplement: Supplementary file 1 — Peer Review File [file 44319_2026_802_MOESM1_ESM.pdf]

EMBO reports

Limitations of the p16-3MR Mouse Model for Detecting and Eliminating Senescent Cells

Nozomi Hori, Shimpei Kawamoto, Ken Uemura, Yumi Kinugasa-Katayama, Yumiko Okumura, Kentaro Tanaka, Jeong Hoon Park, Masahiro Wakita, Daisuke Motooka, Naoko Ohtani, and Eiji Hara

Corresponding author(s): Eiji Hara (ehara@biken.osaka-u.ac.jp) , Shimpei Kawamoto (shimpei.kawamoto@tohoku.ac.jp)

Review Timeline:

Submission Date:	20th Aug 25
Editorial Decision:	6th Dec 25
Revision Received:	3rd Mar 26
Editorial Decision:	24th Mar 26
Revision Received:	26th Mar 26
Accepted:	30th Mar 26

Editor: Bernd Pulverer

Transaction Report:

(Note: With the exception of the correction of typographical or spelling errors that could be a source of ambiguity, letters and reports are not edited. Depending on transfer agreements, referee reports obtained elsewhere may or may not be included in this compilation. Referee reports are anonymous unless the Referee chooses to sign their reports.)

Dear Eiji,

Thank you for the submission of your manuscript to EMBO reports. We have now received the full reports from 2 referees and a short view from a third referee.

As you will see, the referees acknowledge that the findings are potentially important and they are in principle in favour of publication after revision. However, they raise a couple of experimental issues which I highlight here in summary:
Ref 1:

1) 'It would be valuable to know whether bioluminescence can be detected in this controlled setting. Could the authors perform BLI on MEFs at early and late passages? it would be useful to examine whether RFP signal is detectable under the authors' current experimental conditions.'
2) 'Have they also analysed non-carrier littermates from p16-3MR breeding pairs?'
3) 'the transgene may have been functional initially but subsequently became inactivated over generations.'

Ref 2:
1) 'It would be informative to determine whether the model can still be used effectively on dissected organs from aged animals.'
2) 'The utility of the transgene in tissue culture remains of interest: do senescent p16-3MR MEFs emit a stronger signal than wild-type senescent controls?'
3) 'Regarding ganciclovir, further characterisation of its non-specific activity would be valuable:
(i) It would be informative to examine immune cell subsets known to express p16 (B/T cells, macrophages) in different organs of aged wild-type mice following the injection regimen recommended in the original publication.
(ii) Additionally, evaluating p16 mRNA levels in treated old wild-type versus p16-3MR mice could shed light on discrepancies observed between laboratories that have previously used this mouse line under varying conditions.'

ref 3: no specific experimental issues were raised.

In case of refuting papers on key topics in particular, we encourage assembly of as comprehensive a dataset as possible to make a definitive case and ensure the scope of the refutation is clearly delineated.
While I understand that you and your colleagues may be reluctant to invest more time into this project, of the above points, the following are in our view essential:
1) test validity in MEFs (refs 1, 2)
2) test validity in organs form aged animals (ref 2).
3) some further characterization of the non-specific activity
of ganciclovir (ref 2)

Please also comment in the revised manuscript on the hypothesis of ref 1 that the transgene may have been inactivated.

I would thus like to invite you to revise your manuscript partially experimentally and partially textually with the understanding that the referee concerns must be fully addressed and their suggestions taken on board. Please address all referee concerns in a complete point-by-point response.

We realize that it is difficult to revise to a specific deadline. In the interest of protecting the conceptual advance provided by the work, we recommend a revision within 3 months (8th Mar 2026). Please discuss the revision progress ahead of this time with me if you require more time to complete the revisions.

You can either publish the study as a short report or as a full article. For short reports, the revised manuscript should not exceed 27,000 characters (including spaces but excluding materials & methods and references) and 5 main plus 5 expanded view figures. The results and discussion sections must further be combined, which will help to shorten the manuscript text by eliminating some redundancy that is inevitable when discussing the same experiments twice. For a normal article there are no length limitations, but it should have more than 5 main figures and the results and discussion sections must be separate. In both cases, the entire materials and methods must be included in the main manuscript file.

IMPORTANT NOTE: we perform an initial quality control of all revised manuscripts before re-review. Your manuscript will FAIL this control and the handling will be DELAYED if the following APPLIES:
1) A data availability section providing access to data deposited in public databases is missing. If you have not deposited any data, please add a sentence to the data availability section that explains that.
2) Your manuscript contains statistics and error bars based on n=2. Please use scatter blots in these cases. No statistics should be calculated if n=2.

When submitting your revised manuscript, please review the submission guidelines (https://link.springer.com/journal/44319/submission-guidelines#cms-Revised-submissions) and carefully review the instructions that follow below. Failure to include requested items will delay the evaluation of your revision.

1) a .docx formatted version of the manuscript text (including legends for main figures, EV figures and tables). Please make sure that the changes are highlighted to be clearly visible.

2) individual production quality figure files as .eps, .tif, .jpg (one file per figure). See hhttps://media.springernature.com/original/springer-cms/rest/v1/content/27825798/data/v1 for more info on how to prepare your figures.

3) We replaced Supplementary Information with Expanded View (EV) Figures and Tables that are collapsible/expandable online. A maximum of 5 EV Figures can be typeset. EV Figures should be cited as 'Figure EV1, Figure EV2" etc... in the text and their respective legends should be included in the main text after the legends of regular figures.

- For the figures that you do NOT wish to display as Expanded View figures, they should be bundled together with their legends in a single PDF file called *Appendix*, which should start with a short Table of Content. Appendix figures should be referred to in the main text as: "Appendix Figure S1, Appendix Figure S2" etc. See 'Expanded View' section of the submission guidelines.

- Additional Tables/Datasets should be labeled and referred to as Table EV1, Dataset EV1, etc. Legends have to be provided in a separate tab in case of .xls files. Alternatively, the legend can be supplied as a separate text file (README) and zipped together with the Table/Dataset file.

4) a .docx formatted letter INCLUDING the reviewers' reports and your detailed point-by-point responses to their comments. As part of the EMBO Press transparent editorial process, the point-by-point response is part of the Review Process File (RPF), which will be published alongside your paper.

5) a complete author checklist, which you can download from our author guidelines. Please insert information in the checklist that is also reflected in the manuscript. The completed author checklist will also be part of the RPF.

6) Please note that all corresponding authors are required to supply an ORCID ID for their name upon submission of a revised manuscript (), which can be linked to your profile in the manuscript submission system.

7) Before submitting your revision, primary datasets produced in this study need to be deposited in an appropriate public database. Please remember to provide a reviewer password if the datasets are not yet public. The accession numbers and database should be listed in a formal "Data Availability" section placed after Methods. Please note that the Data Availability Section is restricted to new primary data that are part of this study. * Note - All links should resolve to a page where the data can be accessed. *

8) At EMBO Press we ask authors to provide source data for the main manuscript figures. You will receive a separate email with instructions for providing source data with your revised manuscript, including how to upload and organize the files.

9) Our journal also encourages inclusion of *data citations in the reference list* to directly cite datasets that were re-used and obtained from public databases. Data citations in the article text are distinct from normal bibliographical citations and should directly link to the database records from which the data can be accessed. In the main text, data citations are formatted as follows: "Data ref: Smith et al, 2001" or "Data ref: NCBI Sequence Read Archive PRJNA342805, 2017". In the Reference list, data citations must be labeled with "[DATASET]". A data reference must provide the database name, accession number/identifiers and a resolvable link to the landing page from which the data can be accessed at the end of the reference.

10) Regarding data quantification, the following points must be specified in each figure legend:

- the name of the statistical test used to generate error bars and P values,

- the number (n) of independent experiments (please specify technical or biological replicates) underlying each data point,

- the nature of the bars and error bars (s.d., s.e.m.),

- If the data are obtained from n Program fragment delivered error ``Can't locate object method "less" via package "than" (perhaps you forgot to load "than"?) at //ejpvfs23/sites23b/embor_www/letters/embor_decision_revise_and_review_sr_any.txt line 55.' 2, use scatter blots showing the individual data points.

Discussion of statistical methodology can be reported in the materials and methods section, but figure legends should contain a basic description of n, P and the test applied.

- Please also include scale bars in all microscopy images.

11) The journal requires a statement specifying whether or not authors have competing interests (defined as all potential or

actual interests that could be perceived to influence the presentation or interpretation of an article). In case of competing interests, this must be specified in your disclosure statement.

12) All Materials and Methods need to be described in the main text using our 'Structured Methods' format, which is required for all research articles. According to this format, the Methods section includes a Reagents and Tools Table (listing key reagents, experimental models, software and relevant equipment and including their sources and relevant identifiers) followed by a Methods and Protocols section describing the methods using a step-by-step protocol format. The aim is to facilitate adoption of the methodologies across labs. More information on how to adhere to this format as well as a downloadable template (.docx) for the Reagents and Tools Table can be found in our author guidelines.

As part of the EMBO publication's Transparent Editorial Process, EMBO reports publishes online a Review Process File (RPF) to accompany accepted manuscripts. This File will be published in conjunction with your paper and will include the referee reports, your point-by-point response and all pertinent correspondence relating to the manuscript.

I look forward to seeing a revised form of your manuscript when it is ready.

best wishes,

Bernd

~~~~~~~~~~~~~~~~~~~~~~~~~~~~~~~~
Bernd Pulverer, Ph.D.
Chief Editor, EMBO Reports
EMBO
Meyerhofstrasse 1, D-69117 Heidelberg
Tel: +4962218891501
bernd.pulverer@embo.org
~~~~~~~~~~~~~~~~~~~~~~~~~~~~~~~~

Referee #1:

This is a comprehensive and carefully executed study from the Hara Lab, in which the authors re-evaluate several key experiments originally reported in the Demaria et al., (Dev Cell 2014) publication. Two authors of the current submission (NO and EH) were co-authors on that original publication, which presented data using the p16-3MR mouse line to track and ablate p16-expressing senescent cells. The current manuscript submitted to EMBO Reports aims to address important limitations of the p16-3MR model that became apparent after the initial publication in 2014. Notably, despite being authors on the initial Demaria et al. study (2014), neither NO nor EH were involved in the experiments using the p16-3MR mouse line.

The authors begin by examining whether black fur interferes with detection of bioluminescence generated by Renilla luciferase in the p16-3MR line, using mice obtained from the Campisi laboratory. As shown in Figure 1, only very weak luminescent signals are detectable after shaving, and importantly, the same signal intensity is observed in wild-type controls. These observations are consistent with independent reports showing that CTZ-h, the Renilla luciferase substrate, can produce weak luminescence independently of Rluc through reactions with albumin and superoxide. The authors further validate these findings using albino C57BL/6 mice crossed with the p16-3MR line, again demonstrating that only weak, shaving-dependent background signal is detectable.

The manuscript then revisits the increased luminescence previously reported by Demaria et al. (2014) in ageing p16-3MR mice. When comparing p16-3MR and wild-type mice under the same conditions, the authors find no significant differences, indicating that the earlier observations were likely attributable to CTZ-h-derived background noise. Similarly, ganciclovir (GCV) treatment does not reduce bioluminescence in aged mice or following skin injury. Importantly, when these experiments are repeated using the p16-3MR mouse line from the Demaria Lab, the results are essentially identical. Even homozygous p16-3MR mice display the same background signal as heterozygous or wild-type animals.

The authors next test the previously reported increase in bioluminescent signal following doxorubicin-induced DNA damage. In a well-controlled experiment comparing 15 wild-type and 15 p16-3MR mice from the Demaria Lab, treated with PBS or with PBS or GCV following doxorubicin administration, the authors detect no significant differences among any groups. These results further support the conclusion that the reported signals reflect substrate-dependent background rather than true p16-3MR reporter activity.

Given that bioluminescent readouts appear unreliable, the authors then address whether the biological effects of GCV treatment, previously interpreted as senescent cell clearance, might still hold independently of the reporter system. Using MEFs derived

from p16-3MR and wild-type embryos, they demonstrate robust senescence induction with successive passages (as shown by reduced EdU incorporation and increased p16 expression). However, GCV does not selectively eliminate senescent MEFs relative to early-passage controls, nor does doxorubicin sensitise cells to GCV.

Because GCV is known to inhibit proliferation of myeloid cells in a thymidine kinase-independent manner and suppress the cGAS/STING pathway, and given that myeloid cells frequently express high levels of p16, the authors examine whether GCV might reduce macrophage populations independently of the p16-3MR transgene. Indeed, wild-type macrophages exhibit sensitivity to GCV in vitro. This suggests that reductions in senescence markers or SASP factors reported in prior studies may be attributable to these TK-independent effects of GCV rather than transgene-mediated ablation.

Overall, this manuscript provides a rigorous and convincing demonstration of the limitations of the p16-3MR mouse model. It highlights the critical importance of including wild-type controls in any experimental design involving this line and provides valuable clarification for the field.

I have the following suggestions for the authors:

1. Figure 5 is highly informative, as the experiments assessing GCV sensitivity are performed in vitro using MEFs isolated from p16-3MR and wild-type mice. It would be valuable to know whether bioluminescence can be detected in this controlled setting. Could the authors perform BLI on MEFs at early and late passages (i.e., non-senescent vs. senescent MEFs)? This would nicely complement the in vivo analyses and help clarify whether any reporter activity is detectable in vitro.

Similarly, although RFP fluorescence was originally reported to be weak in Demaria et al. (2014), it would be useful to examine whether RFP signal is detectable under the authors' current experimental conditions.

2. The authors use wild-type mice obtained from commercial suppliers. Have they also analysed non-carrier littermates from p16-3MR breeding pairs? Comparing bioluminescence in p16-3MR mice versus their non-carrier littermates would provide an additional, highly controlled assessment of background signal. This could be done in some of the experiments presented in the manuscript using youn mice.

3. One possible explanation for discrepancies between the original publication, subsequent studies, and the present work is that the transgene may have been functional initially but subsequently became inactivated over generations. Transgene silencing is a well-known phenomenon in transgenic mouse lines. Could the authors discuss this possibility in more detail? This does not detract from their central conclusion, namely, that all studies using the p16-3MR line should be carefully interpreted and that proper wild-type controls are essential, but it may help explain why some studies that used p16-3MR and wild-type mice side by side reported different outcomes.

Referee #2:

The study by Hori et al. addresses an important issue regarding the widely used in the past several years p16-3MR transgenic mouse model, which has been employed to detect and eliminate senescent cells. This carefully conducted investigation is to be commended for highlighting potential limitations that could have significant implications for the interpretation of many published findings. Hori et al. identify two major concerns with this model.

First, the authors demonstrate that the Renilla luciferase reporter used in the p16-3MR construct is suboptimal for whole-body imaging of p16 expressing cells, and that a substantial portion of the detected signal may reflect substrate-related background rather than genuine reporter expression. They report that bioluminescence intensity produced by this transgene did not vary when compared to proper negative control mice with advancing age, following doxorubicin exposure, or during cutaneous wound healing-findings that do not replicate earlier observations with this model.

This concern is well founded. Endogenous p16 expression is very low, making the sensitivity of the reporter system critical. Renilla luciferase on the other hand is not only approximately 1-5% as bright as Firefly luciferase, but it also emits predominantly blue-green light, which is strongly absorbed and scattered by tissue and haemoglobin. As a result, signals from deeper organs are heavily attenuated, rendering Renilla suboptimal for whole-body or deep-tissue imaging. Additionally, coelenterazine-based reactions display very rapid kinetics, with signals peaking within seconds to minutes before decaying rapidly. Minor differences in imaging timing can therefore produce substantial variability in measured signal. Taken together, these factors make the use of this transgenic approach to mark senescent cells a legitimate concern.

In light of these technical limitations, Hori et al.'s observation that shaving the animals significantly reduces background and clarifies the signal is entirely convincing. This highlights an often-overlooked pre-analytical variable, and given that many laboratories using p16-3MR mice did not consistently shave animals prior to imaging, the authors' caution is particularly important.

The second major concern relates to the HSV-TK/GCV suicide system, which the authors report does not consistently eliminate senescent cells in vitro or in vivo. Observed effects may, at least in part, be due to non-specific activities of ganciclovir. As ganciclovir has documented off-target and immunosuppressive effects, experiments using systemic administration could yield effects independent of targeted senescent cell ablation unless appropriate controls are included.

While this work represents an important contribution, certain aspects could be further explored to strengthen the conclusions. For example, the limitations of the p16-3MR model for bioluminescence analysis are clear, particularly for non-shaved animals

and whole-body imaging. It would be informative to determine whether the model can still be used effectively on dissected organs from aged animals, or whether haemoglobin presence/other factors as well as rapid signal decay present additional challenges. Similarly, the utility of the transgene in tissue culture remains of interest: do senescent p16-3MR MEFs emit a stronger signal than wild-type senescent controls?
Regarding ganciclovir, further characterisation of its non-specific activity would be valuable. It would be informative to examine immune cell subsets known to express p16 (B/T cells, macrophages) in different organs of aged wild-type mice following the injection regimen recommended in the original publication. Additionally, evaluating p16 mRNA levels in treated old wild-type versus p16-3MR mice could shed light on discrepancies observed between laboratories that have previously used this mouse line under varying conditions.

Ref #3 (general comments):
In my quick review of the two abstracts, I can imagine that each author is partially correct. The substrate coelenterazine is a high energy molecule that is catalyzed by albumin and the stress environment associated with inflammation-the molecule is a good maker of oxidative stress and this is likely why they observe signals in mice without the reporter gene when senescence is induced. In addition the reaction produces blue light which is absorbed by hemoglobin and melanin, so the loss of signal can be significant. The loss of signal and the autocatalysis are likely the cause of the problems in these studies. This seems like a series experiments with flaws due to a lack of understanding.

Point-by-point responses

We sincerely thank the Editor and the Reviewers for their constructive and thoughtful comments, which have greatly helped us improve the manuscript. We believe that addressing these comments has strengthened the study. Below, we provide point-by-point responses to the Editor's and the Reviewers' comments, with our replies shown in bold. We hope that our responses clarify the issues raised and demonstrate that the manuscript merits publication in EMBO Reports.

<u>Editor:</u>

In case of refuting papers on key topics in particular, we encourage assembly of as comprehensive a dataset as possible to make a definitive case and ensure the scope of the refutation is clearly delineated.
While I understand that you and your colleagues may be reluctant to invest more time into this project, of the above points, the following are in our view essential:
1) test validity in MEFs (refs 1, 2)
2) test validity in organs form aged animals (ref 2).
3) some further characterization of the non-specific activity
of ganciclovir (ref 2)
Please also comment in the revised manuscript on the hypothesis of ref 1 that the transgene may have been inactivated.

I would thus like to invite you to revise your manuscript partially experimentally and partially textually with the understanding that the referee concerns must be fully addressed and their suggestions taken on board. Please address all referee concerns in a complete point-by-point response.
We realize that it is difficult to revise to a specific deadline. In the interest of protecting the conceptual advance provided by the work, we recommend a revision within 3 months (8th Mar 2026). Please discuss the revision progress ahead of this time with me if you require more time to complete the revisions.
<u>**Response:**</u>

We sincerely thank the Editor for the thorough evaluation of our manuscript and for the clear and priority guidance on the revisions required. We have performed the additional experiments and explanations requested and revised the manuscript accordingly.

Regarding the point 1, we assessed the validity of the *p16-3MR* model in MEFs by performing bioluminescence imaging (BLI) and immunofluorescence analyses in non-senescent (early-passage, P3) and senescent (late-passage, P8 or doxorubicin-induced) MEFs derived from *p16-3MR* and wild-type mice analyzed side by side. Only weak signals were detected in both genotypes, attributable to substrate (CTZ-h)-dependent background luminescence and senescence-associated cellular autofluorescence. These data are included in Fig. EV2.

Regarding the point 2, a recent bioRxiv preprint from the Demaria group reported *ex vivo* imaging of *p16-3MR* mouse organs after CTZ-h incubation, claiming higher signals in aged mice (Wang *et al.,* 2026). However, based on the presented images (Fig. 1D in Wang et al., 2026), an age-related increase appears limited to the spleen. Moreover, the color bars are too small to determine the detection sensitivity, and WT controls were not included.

Because we do not currently maintain aged *p16-3MR* mice, we were unable to directly test aged animals. Instead, we performed side-by-side *ex vivo* imaging of 5-month-old *p16-3MR* and WT mice using the same protocol. When the signal detection threshold was set to 600 counts—the level recommended by the IVIS system (Revvity Inc.) to avoid background detection—no bioluminescent signals were observed in any organs. Lowering the threshold to 30 counts revealed weak signals; however, no significant differences were detected between *p16-3MR* and WT mice, suggesting substrate-derived background rather than Rluc activity. Given that CTZ-h can generate luminescence through non-enzymatic reactions, results obtained at very low detection thresholds and/or without WT controls should be interpreted with caution. These data are included in Fig. EV3.

Regarding the point 3, because we considered a comprehensive analysis of the effects of ganciclovir (GCV) on immune cell subsets in aged wild-type mice to be beyond the scope of the present study, we expanded the Discussion to highlight reported HSV-TK–independent effects of GCV on macrophages, microglia, and T cell proliferation and function, with reference to relevant prior studies. In addition, using RNA samples from our DXR/GCV treatment experiments (Fig. 4A–C), we assessed endogenous *p16*^{INK4a} mRNA levels in wild-type and *p16-3MR* mice. GCV treatment did not significantly alter *p16*^{INK4a} expression in either genotype (Fig. EV1), further supporting the notion that HSV-TK–dependent effects in *p16-3MR*

mice are limited or absent under these conditions.

Regarding the point 4, we addressed the possibility that the transgene may have been inactivated over generations. We found that the bioluminescence signal intensities reported in Demaria et al. (2014) were already very low and within the noise range of the IVIS system, suggesting that transgene expression was likely minimal even at the time of the original study. Furthermore, side-by-side comparisons of *p16-3MR* mice obtained independently from the Demaria and Campisi laboratories revealed no substantial differences in signal intensity, arguing against a generational decline in transgene activity.

We hope that these revisions address the Editor's essential concerns and demonstrate that the manuscript merits publication in EMBO Reports. Detailed point-by-point responses to each of the Referees' comments are provided below.

Referee #1:

This is a comprehensive and carefully executed study from the Hara Lab, in which the authors re-evaluate several key experiments originally reported in the Demaria et al., (Dev Cell 2014) publication. Two authors of the current submission (NO and EH) were co-authors on that original publication, which presented data using the p16-3MR mouse line to track and ablate p16-expressing senescent cells. The current manuscript submitted to EMBO Reports aims to address important limitations of the p16-3MR model that became apparent after the initial publication in 2014. Notably, despite being authors on the initial Demaria et al. study (2014), neither NO nor EH were involved in the experiments using the p16-3MR mouse line.

The authors begin by examining whether black fur interferes with detection of bioluminescence generated by Renilla luciferase in the p16-3MR line, using mice obtained from the Campisi laboratory. As shown in Figure 1, only very weak luminescent signals are detectable after shaving, and importantly, the same signal intensity is observed in wild-type controls. These observations are consistent with independent reports showing that CTZ-h, the Renilla luciferase substrate, can produce weak luminescence independently of Rluc through reactions with albumin and superoxide. The authors further validate these findings using albino C57BL/6 mice

crossed with the p16-3MR line, again demonstrating that only weak, shaving-dependent background signal is detectable.

The manuscript then revisits the increased luminescence previously reported by Demaria et al. (2014) in ageing p16-3MR mice. When comparing p16-3MR and wild-type mice under the same conditions, the authors find no significant differences, indicating that the earlier observations were likely attributable to CTZ-h-derived background noise. Similarly, ganciclovir (GCV) treatment does not reduce bioluminescence in aged mice or following skin injury. Importantly, when these experiments are repeated using the p16-3MR mouse line from the Demaria Lab, the results are essentially identical. Even homozygous p16-3MR mice display the same background signal as heterozygous or wild-type animals.

The authors next test the previously reported increase in bioluminescent signal following doxorubicin-induced DNA damage. In a well-controlled experiment comparing 15 wild-type and 15 p16-3MR mice from the Demaria Lab, treated with PBS or with PBS or GCV following doxorubicin administration, the authors detect no significant differences among any groups. These results further support the conclusion that the reported signals reflect substrate-dependent background rather than true p16-3MR reporter activity.

Given that bioluminescent readouts appear unreliable, the authors then address whether the biological effects of GCV treatment, previously interpreted as senescent cell clearance, might still hold independently of the reporter system. Using MEFs derived from p16-3MR and wild-type embryos, they demonstrate robust senescence induction with successive passages (as shown by reduced EdU incorporation and increased p16 expression). However, GCV does not selectively eliminate senescent MEFs relative to early-passage controls, nor does doxorubicin sensitise cells to GCV.

Because GCV is known to inhibit proliferation of myeloid cells in a thymidine kinase-independent manner and suppress the cGAS/STING pathway, and given that myeloid cells frequently express high levels of p16, the authors examine whether GCV might reduce macrophage populations independently of the p16-3MR transgene. Indeed, wild-type macrophages exhibit sensitivity to GCV in vitro. This suggests that reductions in senescence markers or SASP factors reported in prior studies may be attributable to these TK-independent effects of GCV rather than transgene-mediated ablation.

Overall, this manuscript provides a rigorous and convincing demonstration of the limitations of the p16-3MR mouse model. It highlights the critical importance of including wild-type controls in any experimental design involving this line and provides valuable clarification for the field.

Response:

We thank Referee #1 for the careful and comprehensive evaluation of our manuscript and for the thoughtful summary of its aims and conclusions. We are grateful for the referee's positive assessment of our study and for recognizing the importance and rigor of our findings.

I have the following suggestions for the authors:

1. Figure 5 is highly informative, as the experiments assessing GCV sensitivity are performed in vitro using MEFs isolated from p16-3MR and wild-type mice. It would be valuable to know whether bioluminescence can be detected in this controlled setting. Could the authors perform BLI on MEFs at early and late passages (i.e., non-senescent vs. senescent MEFs)? This would nicely complement the in vivo analyses and help clarify whether any reporter activity is detectable in vitro.
Similarly, although RFP fluorescence was originally reported to be weak in Demaria et al. (2014), it would be useful to examine whether RFP signal is detectable under the authors' current experimental conditions.

Response:

We thank Referee #1 for this constructive and helpful suggestion. In response to this suggestion, we performed bioluminescence imaging (BLI) and immunofluorescence analyses using cultured MEFs derived from *p16-3MR* and wild-type (WT) mice. We examined both early-passage (P3) non-senescent MEFs and late-passage (P8) or doxorubicin (DXR)-induced senescent MEFs. Under these controlled *in vitro* conditions, neither late-passage senescent MEFs nor DXR-induced senescent MEFs showed increased bioluminescence signals, irrespective of genotype (Fig. EV2A), indicating that no detectable Renilla luciferase-reporter activity was observed by *in vitro* BLI. Notably, bioluminescence signals slightly increased when coelenterazine-h (CTZ-h) was added to cells in DMEM containing fetal bovine serum (FBS), but not in PBS (Fig. EV2A), consistent with previous reports demonstrating that CTZ-h can generate luminescence through reactions

with serum albumin (Zhao et al, 2004).

We also performed immunofluorescence analysis for RFP using these MEFs. Only weak fluorescence signals were detected in senescent MEFs from *p16-3MR* mice; however, similar signals were also observed in WT MEFs, even in the absence of anti-RFP antibody staining (Fig. EV2B). These findings are consistent with a previous report showing that senescent cells exhibit increased auto-fluorescence (Bertolo et al., 2019), suggesting that the detected fluorescence signals likely reflect auto-fluorescence. These new data have been included in Figure EV2 and are described in the revised manuscript (page 12, lines 1-20 in PEF file).

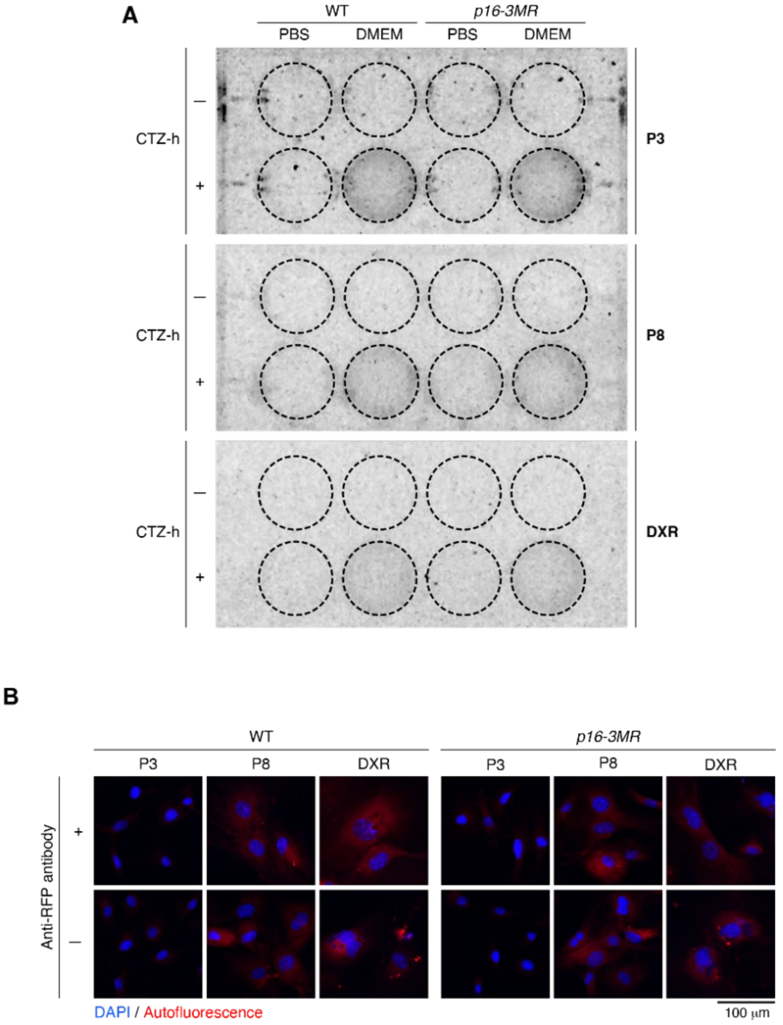

Fig. EV2. Lack of genotype-dependent luminescence and RFP signals in *p16-3MR* MEFs.
(**A**) Representative BLI of early-passage (P3) and senescent (P8 or DXR-treated) MEFs derived from WT or *p16-3MR* embryos. Cellular senescence was induced by serial passaging (P8) or by treatment with doxorubicin (DXR; 100 ng/mL) for 7 days. Prior to imaging, the culture medium was replaced with PBS or DMEM containing 10% FBS, in the presence or absence of CTZ-h (final concentration, 2 µg/mL). (B) Immunofluorescence staining of the indicated MEFs. Cells were stained with or without anti-RFP antibody. Both WT and p16-3MR MEFs at P8 or following DXR treatment exhibited non-specific fluorescence signals (autofluorescence) regardless of the presence of the primary antibody. Experiments were independently repeated at least twice with similar results.

2. The authors use wild-type mice obtained from commercial suppliers. Have they also analysed non-carrier littermates from p16-3MR breeding pairs? Comparing bioluminescence in p16-3MR mice versus their non-carrier littermates would provide an additional, highly controlled assessment of background signal. This could be done in some of the experiments presented in the manuscript using youn mice.

Response:

We thank Referee #1 for this helpful suggestion and apologize for not clearly stating this in the original manuscript. In most cases, the WT mice used in our study were littermates of the *p16-3MR* mice. In particular, the wild-type, heterozygous, and homozygous mice analyzed in Fig. 3D were all littermates derived from the same breeding pairs. We have now clarified this point in the revised manuscript (page 9, line 21 to page 10, line 1 in PDF file).

3. One possible explanation for discrepancies between the original publication, subsequent studies, and the present work is that the transgene may have been functional initially but subsequently became inactivated over generations. Transgene silencing is a well-known phenomenon in transgenic mouse lines. Could the authors discuss this possibility in more detail? This does not detract from their central conclusion, namely, that all studies using the p16-3MR line should be carefully interpreted and that proper wild-type controls are essential, but it may help explain why some studies that used p16-3MR and wild-type mice side by side reported different outcomes.

Response:

We thank Referee #1 for this thoughtful and important comment. We agree that transgene silencing over successive generations is known to occur in some transgenic mouse lines and therefore represents a possibility that warrants consideration. However, it is noteworthy that the bioluminescence signal intensities reported in the original study already appear to be very low at the time of publication (Demaria et al, 2014). Specifically, the reported signal intensities ranged from approximately 10–50 counts (Figure 2A in Demaria et al, 2014), 20–150 counts (Figure 2C in Demaria et al, 2014), 20–80 counts (Figure 3B in Demaria et al, 2014), and 10–50 counts (Figure S2D in Demaria et al, 2014). These values are highly comparable to the signal intensities observed in our current study, as indicated by the color scales shown in both our figures and those of Demaria et al.

(2014), and fall within the noise range according to the user manual of the IVIS imaging system (Revvity Inc.). In addition, we directly compared *p16-3MR* mice obtained from the Demaria laboratory, which were reported to be functional, with those obtained independently from the Campisi laboratory, and analyzed them side by side under identical imaging conditions. We did not observe any substantial differences in bioluminescence signals between the two sources.

Taken together, these observations strongly suggest that the expression level of the *p16-3MR* transgene was very low even at the time of the original publication and appears not to have changed substantially over subsequent generations. We have now discussed these points and their implications more explicitly in the revised manuscript (page 10, lines 4-19 in PDF file), and we thank the referee for prompting this important clarification.

Referee #2:

The study by Hori et al. addresses an important issue regarding the widely used in the past several years p16-3MR transgenic mouse model, which has been employed to detect and eliminate senescent cells. This carefully conducted investigation is to be commended for highlighting potential limitations that could have significant implications for the interpretation of many published findings. Hori et al. identify two major concerns with this model.

First, the authors demonstrate that the Renilla luciferase reporter used in the p16-3MR construct is suboptimal for whole-body imaging of p16 expressing cells, and that a substantial portion of the detected signal may reflect substrate-related background rather than genuine reporter expression. They report that bioluminescence intensity produced by this transgene did not vary when compared to proper negative control mice with advancing age, following doxorubicin exposure, or during cutaneous wound healing-findings that do not replicate earlier observations with this model.

This concern is well founded. Endogenous p16 expression is very low, making the sensitivity of the reporter system critical. Renilla luciferase on the other hand is not only approximately 1-5% as bright as Firefly luciferase, but it also emits predominantly blue-green light, which is strongly absorbed and scattered by tissue and haemoglobin. As a result, signals from deeper organs are heavily attenuated, rendering Renilla suboptimal for whole-body or deep-tissue imaging. Additionally, coelenterazine-based reactions display very rapid kinetics, with signals peaking within seconds to minutes before

decaying rapidly. Minor differences in imaging timing can therefore produce substantial variability in measured signal. Taken together, these factors make the use of this transgenic approach to mark senescent cells a legitimate concern.

In light of these technical limitations, Hori et al.'s observation that shaving the animals significantly reduces background and clarifies the signal is entirely convincing. This highlights an often-overlooked pre-analytical variable, and given that many laboratories using p16-3MR mice did not consistently shave animals prior to imaging, the authors' caution is particularly important.

The second major concern relates to the HSV-TK/GCV suicide system, which the authors report does not consistently eliminate senescent cells in vitro or in vivo. Observed effects may, at least in part, be due to non-specific activities of ganciclovir. As ganciclovir has documented off-target and immunosuppressive effects, experiments using systemic administration could yield effects independent of targeted senescent cell ablation unless appropriate controls are included.

Response:

We thank Referee #2 for the careful reading of our manuscript and for the thoughtful and balanced assessment of our study. We appreciate the referee's recognition of the technical limitations of the *p16-3MR* model, including the constraints of Renilla luciferase–based imaging and the potential non-specific effects of ganciclovir, and the relevance of our findings for the field.

While this work represents an important contribution, certain aspects could be further explored to strengthen the conclusions. For example, the limitations of the p16-3MR model for bioluminescence analysis are clear, particularly for non-shaved animals and whole-body imaging. It would be informative to determine whether the model can still be used effectively on dissected organs from aged animals, or whether haemoglobin presence/other factors as well as rapid signal decay present additional challenges.

Response:

We thank Referee #2 for this insightful suggestion. Recently, the Demaria group reported *ex vivo* imaging data in a bioRxiv preprint using an organ incubation method in which dissected organs were incubated in CTZ-h solution for 45–60 minutes (Wang et al, 2026).

https://www.biorxiv.org/content/10.64898/2026.01.28.701695v1.full

The authors presented imaging data from young and aged *p16-3MR* mice and reported higher bioluminescent signals in aged mice across multiple organs.

However, careful examination of the presented images (Fig. 1D in Wang et al, 2026) suggests that a clearly higher signal in aged mice is apparent only in the spleen. Furthermore, the color bars shown in the images are too small to allow determination of the signal detection sensitivity (cutoff threshold). Importantly, these data did not include wild-type (WT) control animals.

Unfortunately, we do not currently maintain sufficiently aged *p16-3MR* mice and were therefore unable to directly evaluate the reproducibility of these findings in aged animals. However, because we had access to 5-month-old *p16-3MR* and WT mice, we performed side-by-side *ex vivo* imaging of *p16-3MR* and WT mice using the same experimental protocol described by Wang *et al.*
Under these conditions, when the signal detection threshold was set to 600 counts—a level recommended by the IVIS system (Revvity Inc.) as a reliable threshold to avoid detection of background noise—no bioluminescent signals were detected in any organs. In contrast, when the detection threshold was lowered to an extremely low level (30 counts), similar to that used in Demaria *et al* (2014), weak signals became detectable in multiple organs. However, no significant differences were observed between organs from *p16-3MR* and WT mice (Fig. EV3). These observations suggest that the detected luminescence most likely reflects substrate (CTZ-h)-dependent background rather than Rluc activity.

As discussed in our manuscript, CTZ-h is known to generate weak luminescent signals through reactions with serum albumin or superoxide. Therefore, data obtained at detection thresholds set substantially below the level recommended by the IVIS system (Revvity Inc.), and/or in the absence of WT controls, should be interpreted with caution. These new data have been included in Figure EV3 and are described in the revised manuscript (page 13, line 21 to page 14, line 9 in PDF file).

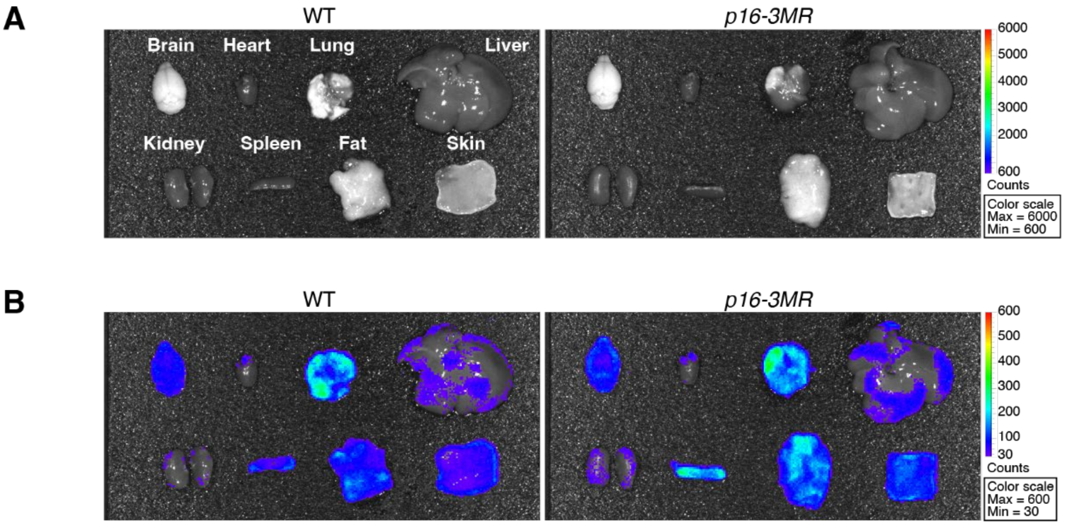

Fig. EV3. No significant difference in ex vivo bioluminescence in organs between *p16-3MR* and WT mice. (**A, B**) Organs were harvested from three independent 5-month-old WT and *p16-3MR* mice and incubated in CTZ-h diluted 1:10 in PBS (final concentration, 15 µg/mL) for 45–60 min according to the protocol described by Wang et al. (2026). *Ex vivo* bioluminescence imaging was performed using an IVIS imaging system. The same imaging data are displayed using different color scale ranges: minimum and maximum thresholds were set to 600 and 6,000 counts in (**A**) and to 30 and 600 counts in (**B**). The binning setting was "medium" for all acquisitions. Color bars represent signal intensity (counts) corresponding to the indicated minimum and maximum thresholds.

Similarly, the utility of the transgene in tissue culture remains of interest: do senescent p16-3MR MEFs emit a stronger signal than wild-type senescent controls?

<u>Response:</u>

We thank Referee #2 for raising this point regarding the utility of the *p16-3MR* transgene in tissue culture. This issue was also raised by Referee #1, and we have addressed it in detail in our response to Referee #1, point 1, where we present additional bioluminescence imaging and fluorescence analyses using senescent and non-senescent MEFs derived from *p16-3MR* and wild-type mice.

Briefly, under our experimental conditions, senescent *p16-3MR* MEFs did not show increased bioluminescence signals compared with wild-type senescent controls, and the detected signals were attributable to substrate (CTZ-h)-dependent background luminescence and autofluorescence (Fig. EV2).

Regarding ganciclovir, further characterisation of its non-specific activity would be valuable. It would be informative to examine immune cell subsets known to express p16 (B/T cells, macrophages) in different organs of aged wild-type mice following the injection regimen recommended in the original publication.

Response:

We thank Referee #2 for this important comment regarding the non-specific effects of ganciclovir. We agree that further characterization of its HSV-TK–independent impact on immune cell populations, including p16-expressing immune subsets in aged wild-type mice, would be informative. However, such analyses would require extensive additional experimentation, including comprehensive organ-wide profiling of immune cell subsets in aged animals, and therefore fall outside the primary focus of the present study, which was to determine whether phenotypes previously reported for *p16-3MR*–mediated senescent cell ablation can be reliably reproduced under our experimental conditions. In this regard, the impact of ganciclovir on immune cells has been reported previously, including studies demonstrating inhibitory effects on macrophages, microglia, and T cell proliferation and function independent of HSV-TK expression (e.g., PMID: 24493798; PMID: 17580254). We have therefore cited the relevant literature and expanded the Discussion to highlight ganciclovir's known non-specific effects as an important caveat when interpreting experiments involving systemic ganciclovir administration (page 12, line 21 to page 13, line 15 in PDF file).

Additionally, evaluating p16 mRNA levels in treated old wild-type versus p16-3MR mice could shed light on discrepancies observed between laboratories that have previously used this mouse line under varying conditions.

Response:

We thank Referee #2 for this thoughtful and valuable suggestion. We agree that evaluating endogenous p16 mRNA levels in treated aged wild-type and *p16-3MR* mice would help clarify discrepancies reported between laboratories. However, generating additional cohorts of aged *p16-3MR* mice and performing the required treatments would require substantial additional time and resources and was therefore not feasible within the timeframe of the present revision.

As an alternative approach, we took advantage of experiments already performed in this study and the corresponding stored RNA samples. In a follow-up study using *p16-3MR* mice, Demaria et al (2017). reported that doxorubicin (DXR) treatment increased endogenous *p16* expression in skin, liver, and lung tissues, and that subsequent ganciclovir treatment reduced endogenous *p16* expression in the lung (Figs. 1G and 2B in Demaria et al, 2017). In our study, we administered DXR

and ganciclovir side by side to wild-type and *p16-3MR* mice (Fig. 4A–C) to assess whether these findings could be reproduced. Using tissues obtained from these experiments, we further examined endogenous p16 mRNA levels. As shown in Fig. EV1, we did not observe significant changes in endogenous p16 expression in *p16-3MR* mice, with no genotype-dependent differences compared with wild-type controls. These results further suggest that the *3MR* transgene does not exhibit detectable functional activity in *p16-3MR* mice under our experimental conditions. The corresponding data are described in the revised text (page 11, lines 3-5 in PDF file).

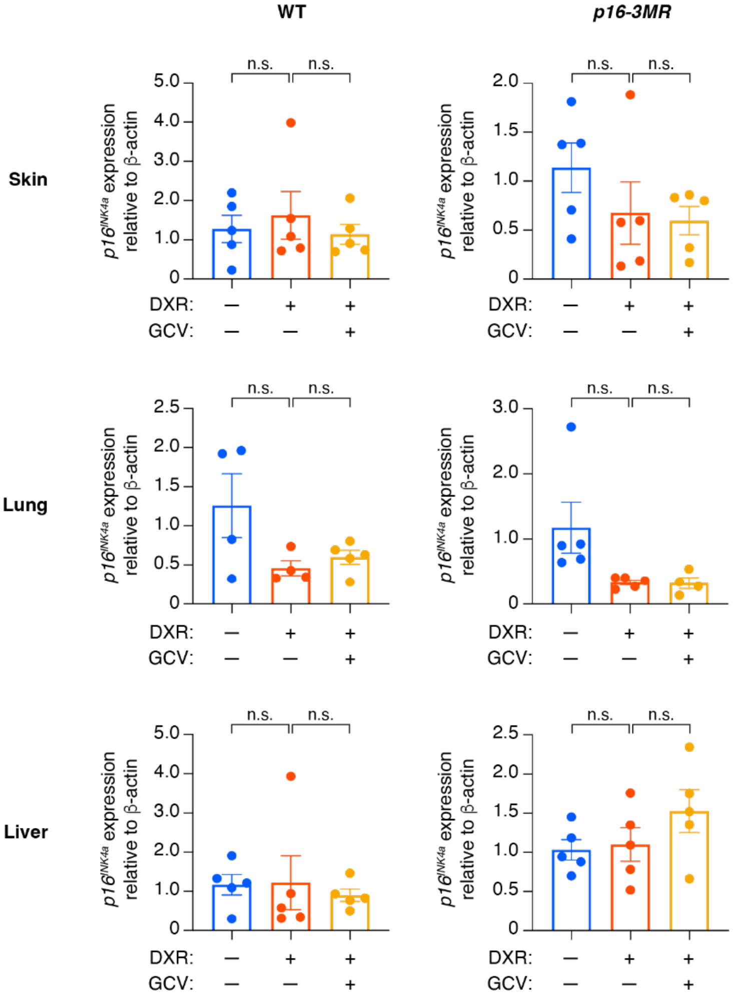

Fig. EV1. No change in *p16*^{INK4a} expression in *p16-3MR* tissues after DXR or GCV treatment.
Three-month-old female WT and *p16-3MR* mice obtained from the Demaria laboratory (the same cohort used in Fig. 4A–C) were intraperitoneally injected with PBS (vehicle control) or doxorubicin (DXR; 10 mg/kg) on day 0. From days 5 to 9, mice received daily intraperitoneal injections of PBS (vehicle control) or ganciclovir (GCV; 25 mg/kg). On day 10, *p16*^{INK4a} expression levels in the liver, skin, and lung were analyzed by RT–qPCR. Data are presented as relative *p16*^{INK4a} expression normalized to the untreated control group (DXR−/GCV−). The sample size (n) represents the number of biological replicates (n = 4–5). Data are presented as mean ± s.e.m. Statistical significance was determined by one-way ANOVA followed by Šídák's multiple-comparison test. n.s., not significant. Experiments were independently repeated at least twice with similar results.

Ref #3 (general comments):

In my quick review of the two abstracts, I can imagine that each author is partially correct. The substrate coelenterazine is a high energy molecule that is catalyzed by albumin and the stress environment associated with inflammation-the molecule is a good maker of oxidative stress and this is likely why they observe signals in mice without the reporter gene when senescence is induced. In addition the reaction produces blue light which is absorbed by hemoglobin and melanin, so the loss of signal can be significant. The loss of signal and the autocatalysis are likely the cause of the problems in these studies. This seems like a series experiments with flaws due to a lack of understanding.

Response:

We thank Referee #3 for these insightful comments and for highlighting the biochemical and optical properties of coelenterazine-based bioluminescence. We agree that substrate-dependent reactions and tissue absorption can strongly influence signal detection and may have contributed to misinterpretation of signals in earlier studies. A central aim of our study was to experimentally evaluate how these factors affect the interpretation of *p16-3MR*–based readouts and to clarify the resulting technical limitations of this mouse model.

1st Revision - Editorial Decision 24th Mar 2026

Manuscript number: EMBOR-2025-62577V2
Title: Limitations of the p16-3MR Mouse Model in Detecting and Eliminating Senescent Cells: A Case Study Revealing the Need for Rigorous Validation of Senescence Models
Author(s): Eiji Hara, Nozomi Hori, Shimpei Kawamoto, Ken Uemura, Yumi Katayama, Yumiko Okumura, Kentaro Tanaka, Jeong Hoon Park, Masahiro Wakita, Daisuke Motooka, and Naoko Ohtani

Dear Eiji

Thank you for your patience while we re-reviewed your revised manuscript. As you will see from the reports below, the referees are both positive and recommend publication in EMBO reports, noting 'the work now clearly highlights important limitations of the p16-3MR mouse model' and 'I commend the authors for bringing such an important issue to the attention of the scientific community'.

We will be glad to publish the manuscript following a few minor issues:

1) The title is too long. We suggest 'Limitations of the p16-3MR Mouse Model for Detecting and Eliminating Senescent Cells'
2) Funding: there is no grant number for Mitsubishi foundation - please confirm if that is intentional
3) We seem to be missing the synopsis text (a short summary and bullet points designed to summarize key finding in the paper in a way that is complementary to the abstract). Please also confirm a synopsis image/visual abstract has been supplied.
4) Figures 3A and 3B are referred to before Fig. 2E, F in the main text: please check to confirm and adjust as necessary
5) Please confirm all relevant source data has been supplied. The data availability section could mention source data has been provided and list relevant fig panels if it is for a subset of figures.

Once you have made these minor revisions, please use the following link to submit your corrected manuscript:

Link Not Available

Thank you for your valuable contribution to EMBO reports.

I should note again that we had given Dr. Demaria the opportunity to frame a short response for co-review and potential publication alongside your paper.

best wishes,

Bernd

~~~~~~~~~~~~~~~~~~~~~~~~~~~~~~~~
Bernd Pulverer, Ph.D.
Chief Editor, EMBO Reports
EMBO
Meyerhofstrasse 1, D-69117 Heidelberg
Tel: +4962218891501
bernd.pulverer@embo.org
~~~~~~~~~~~~~~~~~~~~~~~~~~~~~~~~

Referee #1:

I am satisfied with the authors' response to my previous comments. The additional experiments strengthen the manuscript and further support its main conclusions. In particular, the work now clearly highlights important limitations of the p16-3MR mouse model and underscores the need to include appropriate wild-type control mice lacking the p16-3MR allele in such studies.
I have no further comments.

Referee #2:

This is a revised version of the manuscript that I previously reviewed for potential publication in EMBO Reports. The authors have addressed all of my previous concerns satisfactorily, including the addition of relevant data in the supplementary materials. I now find the manuscript suitable for publication.
I would also like to commend the authors for bringing such an important issue to the attention of the scientific community.

Point-by-point response

Thank you very much for your careful evaluation of our manuscript and for your helpful editorial comments. We have revised the manuscript accordingly and addressed all of the points raised. Our point-by-point responses to the editorial requests are provided below, with our responses indicated **in bold.**

---

1) The title is too long. We suggest 'Limitations of the p16-3MR Mouse Model for Detecting and Eliminating Senescent Cells'

Response:

In line with the editor's suggestion, we have changed the title as follows: "Limitations of the p16-3MR Mouse Model for Detecting and Eliminating Senescent Cells".

---

2) Funding: there is no grant number for Mitsubishi foundation - please confirm if that is intentional

Response:

We have added the grant number for the Mitsubishi Foundation, as well as additional grant information with corresponding grant numbers.

---

3) We seem to be missing the synopsis text (a short summary and bullet points designed to summarize key findings in the paper in a way that is complementary to the abstract). Please also confirm a synopsis image/visual abstract has been supplied.

Response:

We have now uploaded the synopsis text and bullet points. A visual abstract has also been provided.

---

4) Figures 3A and 3B are referred to before Fig. 2E, F in the main text: please check to confirm and adjust as necessary.

Response:

We apologize for any confusion. Figures 3A and 3B referred to before Fig. 2E and 2F correspond to those in Demaria et al. (2014), rather than this manuscript. To avoid confusion, we have revised the text as follows: "as reported in Demaria et al. (2014) (their Figs. 3A and 3B)".

---

5) Please confirm all relevant source data has been supplied. The data availability section could mention source data has been provided and list relevant fig panels if it is for a subset of figures.

<u>Response:</u>

We have confirmed that all relevant source data have been provided and have revised the Data Availability section as follows: "Source data underlying Figs. 2B, 2F, 3C, 3E, 4C, 4F, 5A, 5B, 5C, 5D, 5F and 5G are provided as Source Data files."

In addition to the above points, we have also revised the following:

i) Regarding the acknowledgements, we have clarified the wording to avoid potential confusion, as follows:

"We are grateful to Dr. J Campisi for valuable discussions at the early stages of this work, …"

---

ii) Regarding the tone of the manuscript, we fully agree with your suggestion that statements which could be perceived as overly strong or evaluative should be toned down. Accordingly, in addition to removing the passage you indicated ("Because aging research typically requires long-term studies …"), we have also removed the preceding sentences that could be interpreted as suggesting evaluative judgment about experimental practice ("Importantly, however, adherence to fundamental principles of experimental design … reflects insufficient experimental validation"), to ensure a consistently neutral tone.

As these passages appeared at the end of the manuscript, we have instead concluded the paper with the following statement to maintain an appropriate and balanced ending:

"Taken together, our findings demonstrate that bioluminescence signals detected in the p16-3MR model are most consistent with substrate-dependent background under the conditions tested, and do not reliably reflect endogenous *$p16^{INK4a}$* expression. These results

underscore the need for careful interpretation of studies using the *p16-3MR* model, particularly in aging and senolytics research."

---

iii) Regarding the sentence referring to discussions among co-authors, we agree that this wording could potentially cause confusion for readers, and we have therefore removed it.

Prof. Eiji Hara
The University of Osaka
Institute for Microbial Diseases
3-1, Yamada-oka
Suita
Osaka 565-0871
Japan

Dear Prof. Hara,

I am very pleased to accept your manuscript for publication in the next available issue of EMBO reports. Thank you for your contribution to our journal.

Your manuscript will be processed for publication by EMBO Press. It will be copy edited and you will receive page proofs prior to publication. Please note that you will be contacted by Springer Nature Author Services to complete licensing and payment information.

If you have any questions, please do not hesitate to contact the Editorial Office. Thank you for your contribution to EMBO Reports.

Yours sincerely,

Bernd Pulverer

~~~~~~~~~~~~~~~~~~~~~~~~~~~~~~~~
Bernd Pulverer, Ph.D.
Chief Editor, EMBO Reports
EMBO
Meyerhofstrasse 1, D-69117 Heidelberg
Tel: +4962218891501
bernd.pulverer@embo.org
~~~~~~~~~~~~~~~~~~~~~~~~~~~~~~~~

>>> Please note that it is EMBO Reports policy for the transcript of the editorial process (containing referee reports and your response letter) to be published as an online supplement to each paper. If you do NOT want this, you will need to inform the Editorial Office via email immediately. More information is available here: https://link.springer.com/partners/embo-press/editorial-policies#Peer%20review

EMBO Press Author Checklist

Corresponding Author Name: Eiji Hara
Journal Submitted to: EMBO reports
Manuscript Number: EMBOR-2025-62577V1

USEFUL LINKS FOR COMPLETING THIS FORM
The EMBO Journal - Author Guidelines
EMBO Reports - Author Guidelines
Molecular Systems Biology - Author Guidelines
EMBO Molecular Medicine - Author Guidelines

Reporting Checklist for Life Science Articles (updated January
This checklist is adapted from Materials Design Analysis Reporting (MDAR) Checklist for Authors. MDAR establishes a minimum set of requirements in transparent reporting in the life sciences (see Statement of Task: 10.31222/osf.io/9sm4x). Please follow the journal's guidelines in preparing your
Please note that a copy of this checklist will be published alongside your article.

Abridged guidelines for figures
1. Data
The data shown in figures should satisfy the following conditions:
- the data were obtained and processed according to the field's best practice and are presented to reflect the results of the experiments in an accurate and unbiased manner.
- ideally, figure panels should include only measurements that are directly comparable to each other and obtained with the same assay.
- plots include clearly labeled error bars for independent experiments and sample sizes. Unless justified, error bars should not be shown for technical
- if n<5, the individual data points from each experiment should be plotted. Any statistical test employed should be justified.
- Source Data should be included to report the data underlying figures according to the guidelines set out in the authorship guidelines on Data

2. Captions
Each figure caption should contain the following information, for each panel where they are relevant:
- a specification of the experimental system investigated (eg cell line, species name).
- the assay(s) and method(s) used to carry out the reported observations and measurements.
- an explicit mention of the biological and chemical entity(ies) that are being measured.
- an explicit mention of the biological and chemical entity(ies) that are altered/varied/perturbed in a controlled manner.
- the exact sample size (n) for each experimental group/condition, given as a number, not a range;
- a description of the sample collection allowing the reader to understand whether the samples represent technical or biological replicates (including how many animals, litters, cultures, etc.).
- a statement of how many times the experiment shown was independently replicated in the laboratory.
- definitions of statistical methods and measures:
  - common tests, such as t-test (please specify whether paired vs. unpaired), simple $\chi 2$ tests, Wilcoxon and Mann-Whitney tests, can be unambiguously identified by name only, but more complex techniques should be described in the methods section;
  - are tests one-sided or two-sided?
  - are there adjustments for multiple comparisons?
  - exact statistical test results, e.g., P values = x but not P values < x;
  - definition of 'center values' as median or average;
  - definition of error bars as s.d. or s.e.m.

Please complete ALL of the questions below.
Select "Not Applicable" only when the requested information is not relevant for your study.

Materials

Newly Created Materials	Information included in the manuscript?	In which section is the information available? (Reagents and Tools Table, Materials and Methods, Figures, Data Availability Section)
New materials and reagents need to be available; do any restrictions apply?	Not Applicable	

Antibodies	Information included in the manuscript?	In which section is the information available? (Reagents and Tools Table, Materials and Methods, Figures, Data Availability Section)
For **antibodies** provide the following information:<br>- Commercial antibodies: RRID (if possible) or supplier name, catalogue number and or/clone number<br>- Non-commercial: RRID or citation	Yes	Reagents and Tools Table

DNA and RNA sequences	Information included in the manuscript?	In which section is the information available? (Reagents and Tools Table, Materials and Methods, Figures, Data Availability Section)
**Short novel DNA or RNA including primers, probes**: provide the sequences.	Yes	Reagents and Tools Table

Cell materials	Information included in the manuscript?	In which section is the information available? (Reagents and Tools Table, Materials and Methods, Figures, Data Availability Section)
**Cell lines:** Provide species information, strain. Provide accession number in repository **OR** supplier name, catalog number, clone number, and/**OR** RRID.	Not Applicable	
**Primary cultures:** Provide species, strain, sex of origin, genetic modification status.	Yes	Methods
Report if the cell lines were recently **authenticated** (e.g., by STR profiling) and tested for mycoplasma contamination.	Not Applicable	

Experimental animals	Information included in the manuscript?	In which section is the information available? (Reagents and Tools Table, Materials and Methods, Figures, Data Availability Section)
**Laboratory animals or Model organisms:** Provide species, strain, sex, age, genetic modification status. Provide accession number in repository **OR** supplier name, catalog number, clone number, **OR** RRID.	Yes	Reagents and Tools Table, Methods, Figure legend
**Animal observed in or captured from the field:** Provide species, sex, and age where possible.	Not Applicable	
Please detail **housing and husbandry conditions**.	Yes	Methods

Plants and microbes	Information included in the manuscript?	In which section is the information available? (Reagents and Tools Table, Materials and Methods, Figures, Data Availability Section)
**Plants:** provide species and strain, ecotype and cultivar where relevant, unique accession number if available, and source (including location for collected wild specimens).	Not Applicable	
**Microbes:** provide species and strain, unique accession number if available, and source.	Not Applicable	

Human research participants	Information included in the manuscript?	In which section is the information available? (Reagents and Tools Table, Materials and Methods, Figures, Data Availability Section)
If collected and within the bounds of privacy constraints report on age, sex and gender or ethnicity for all study participants.	Not Applicable	

Core facilities	Information included in the manuscript?	In which section is the information available? (Reagents and Tools Table, Materials and Methods, Figures, Data Availability Section)
If your work benefited from core facilities, was their service mentioned in the acknowledgments section?	Yes	Methods

Design

Study protocol	Information included in the manuscript?	In which section is the information available? (Reagents and Tools Table, Materials and Methods, Figures, Data Availability Section)
If study protocol has been **pre-registered, provide DOI in the manuscript**. For clinical trials, provide the trial registration number **OR** cite DOI.	Not Applicable	
Report the **clinical trial registration number** (at ClinicalTrials.gov or equivalent), where applicable.	Not Applicable	

Laboratory protocol	Information included in the manuscript?	In which section is the information available? (Reagents and Tools Table, Materials and Methods, Figures, Data Availability Section)
Provide DOI OR other citation details if **external detailed step-by-step protocols** are available.	Not Applicable	

Experimental study design and statistics	Information included in the manuscript?	In which section is the information available? (Reagents and Tools Table, Materials and Methods, Figures, Data Availability Section)
Include a statement about **sample size** estimate even if no statistical methods were used.	Yes	Methods
Were any steps taken to minimize the effects of subjective bias when allocating animals/samples to treatment (e.g. **randomization procedure**)? If yes, have they been described?	Yes	Methods
Include a statement about **blinding** even if no blinding was done.	Yes	Methods
Describe **inclusion/exclusion criteria** if samples or animals were excluded from the analysis. Were the criteria pre-established? If sample or data points were omitted from analysis, report if this was due to attrition or intentional exclusion and provide justification.	Yes	Methods
For every figure, are **statistical tests** justified as appropriate? Do the data meet the assumptions of the tests (e.g., normal distribution)? Describe any methods used to assess it. Is there an estimate of variation within each group of data? Is the variance similar between the groups that are being statistically compared?	Yes	Figures

Sample definition and in-laboratory replication	Information included in the manuscript?	In which section is the information available? (Reagents and Tools Table, Materials and Methods, Figures, Data Availability Section)
In the figure legends: state number of times the experiment was **replicated** in laboratory.	Yes	Figures
In the figure legends: define whether data describe **technical or biological replicates**.	Yes	Figures

Ethics

Ethics	Information included in the manuscript?	In which section is the information available? (Reagents and Tools Table, Materials and Methods, Figures, Data Availability Section)
Studies involving **human participants**: State details of **authority granting ethics approval** (IRB or equivalent committee(s), provide reference number for approval.	Not Applicable	
Studies involving **human participants**: Include a statement confirming that **informed consent** was obtained from all subjects and that the experiments conformed to the principles set out in the WMA Declaration of Helsinki and the Department of Health and Human Services Belmont Report.	Not Applicable	
Studies involving **human participants:** For publication of **patient photos**, include a statement confirming that consent to publish was obtained.	Not Applicable	
Studies involving experimental **animals**: State details of **authority granting ethics approval** (IRB or equivalent committee(s), provide reference number for approval. Include a statement of compliance with ethical regulations.	Yes	Methods
Studies involving **specimen and field samples:** State if relevant **permits** obtained, provide details of authority approving study; if none were required, explain why.	Not Applicable	

Dual Use Research of Concern (DURC)	Information included in the manuscript?	In which section is the information available? (Reagents and Tools Table, Materials and Methods, Figures, Data Availability Section)
Could your study fall under dual use research restrictions? Please check biosecurity documents and list of **select agents and toxins** (CDC): https://www.selectagents.gov/sat/list.htm	Not Applicable	
If you used a select agent, is the security level of the lab appropriate and reported in the manuscript?	Not Applicable	
If a study is subject to dual use research of concern regulations, is the name of the **authority granting approval and reference number** for the regulatory approval provided in the manuscript?	Not Applicable	

Reporting

The MDAR framework recommends adoption of discipline-specific guidelines, established and endorsed through community initiatives. Journals have their own policy about requiring specific guidelines and recommendations to complement MDAR.

Adherence to community standards	Information included in the manuscript?	In which section is the information available? (Reagents and Tools Table, Materials and Methods, Figures, Data Availability Section)
State if relevant guidelines or checklists (e.g., **ICMJE, MIBBI, ARRIVE, PRISMA**) have been followed or provided.	Not Applicable	
For **tumor marker prognostic studies,** we recommend that you follow the **REMARK** reporting guidelines (see link list at top right). See author guidelines, under 'Reporting Guidelines'. Please confirm you have followed these guidelines.	Not Applicable	
For **phase II and III randomized controlled trials**, please refer to the **CONSORT** flow diagram (see link list at top right) and submit the CONSORT checklist (see link list at top right) with your submission. See author guidelines, under 'Reporting Guidelines'. Please confirm you have submitted this list.	Not Applicable	

Data Availability

Data availability	Information included in the manuscript?	In which section is the information available? (Reagents and Tools Table, Materials and Methods, Figures, Data Availability Section)
Have **primary datasets** been deposited according to the journal's guidelines (see 'Data Deposition' section) and the respective accession numbers provided in the Data Availability Section?	Not Applicable	
Were **human clinical and genomic datasets** deposited in a public access-controlled repository in accordance to ethical obligations to the patients and to the applicable consent agreement?	Not Applicable	
Are **computational models** that are central and integral to a study available without restrictions in a machine-readable form? Were the relevant accession numbers or links provided?	Not Applicable	
If publicly available data were reused, provide the respective **data citations in the reference list**.	Not Applicable	
